# MXenes-polymer nanocomposites for biomedical applications: fundamentals and future perspectives

**DOI:** 10.3389/fchem.2024.1400375

**Published:** 2024-05-28

**Authors:** D. Parajuli

**Affiliations:** Research Center for Applied Science and Technology, Tribhuvan University, Kathmandu, Nepal

**Keywords:** MXene-polymers nanocomposites, biomedical applications, biocompatibility and toxicity, drug delivery and targeting, cost effective

## Abstract

The article discusses the promising synergy between MXenes and polymers in developing advanced nanocomposites with diverse applications in biomedicine domains. MXenes, possessing exceptional properties, are integrated into polymer matrices through various synthesis and fabrication methods. These nanocomposites find applications in drug delivery, imaging, diagnostics, and environmental remediation. They offer improved therapeutic efficacy and reduced side effects in drug delivery, enhanced sensitivity and specificity in imaging and diagnostics, and effectiveness in water purification and pollutant removal. The perspective also addresses challenges like biocompatibility and toxicity, while suggesting future research directions. In totality, it highlights the transformative potential of MXenes-polymer nanocomposites in addressing critical issues across various fields.

## 1 Introduction

MXenes are a family of two-dimensional (2D) transition metal carbides, nitrides, or carbonitrides that exhibit a unique combination of properties, making them highly versatile materials (Naguib et al., 2014). MXenes are derived from layered ternary carbide, nitride, or carbonitride precursors known as MAX phases. The structure involves selective etching (Ghidiu et al., 2014) of the “A” element layers (typically aluminum) from the MAX phase, leaving behind a 2D layered structure. The general formula for MXenes is M_n+1_X_n_T_x_, where M represents a transition metal (e.g., titanium, tantalum, or niobium), X is carbon and/or nitrogen, T represents surface terminations (such as hydroxyl or fluorine), and n is the number of metal layers (Halim et al., 2014). Without the hydroxyl or oxygen termination, they are hydrophilic (Harris et al., 2015; Halim, Cook, et al., 2016). MXenes typically exhibit metallic conductivity, making them suitable for applications in electronics, sensors (Guo et al., 2019), and energy storage devices (Simon and Gogotsi, 2008; Meng et al., 2014; Kurra, Hota, and Alshareef, 2015). The presence of transition metal layers contributes to their excellent electronic conductivity. MXenes possess impressive mechanical properties, including high stiffness and strength, making them suitable for applications where mechanical integrity is crucial (Ling et al., 2014). MXenes are chemically stable in various environments, showing feasibility for oxidation and resistance to corrosion. MXenes and their oxides are promising topological materials that are experimentally and computationally studied (Parajuli and Samatha, 2022; 2019; Parajuli, Kaphle, and Samatha, 2019; Parajuli and Samatha, 2022; Parajuli, Uppugalla, et al., 2023) (S. Huang and Mochalin, 2019)(S. Huang and Mochalin, 2019). This stability contributes to their applicability in a wide range of conditions. The surface of MXenes is rich in functional groups, such as hydroxyl (OH-) and/or fluorine (F-) terminations, providing reactive sites for further functionalization. Surface functionalization enhances their compatibility with other materials and expands their application range (Harris et al., 2015; Halim, Cook, et al., 2016). MXenes typically have a high specific surface area due to their layered structure, which is advantageous for applications such as energy storage, catalysis, and sensing. MXenes exhibit interesting optical properties, including strong light absorption, which can be exploited in various optoelectronic and sensing applications. Their incorporation for energy storage were found more efficient that those we studied earlier (Parajuli, Murali, Samatha, and Veeraiah, 2022; Parajuli, Taddesse, Murali, Veeraiah, et al., 2022). MXenes demonstrate good thermal conductivity, making them promising candidates for applications in thermal management and electronic devices (Forrest, 2004; Y; Shi et al., 2015; Reese et al., 2004). The unique combination of electronic, mechanical, chemical, and optical properties makes MXenes a promising class of materials with diverse applications in fields ranging from electronics and energy storage to catalysis and biomedicine (Z. Liu et al., 2018; C; Dai, Chen, et al., 2017; Lin et al., 2018; X; Han et al., 2018) (C. Dai, Lin, et al., 2017).

**FIGURE 1:**
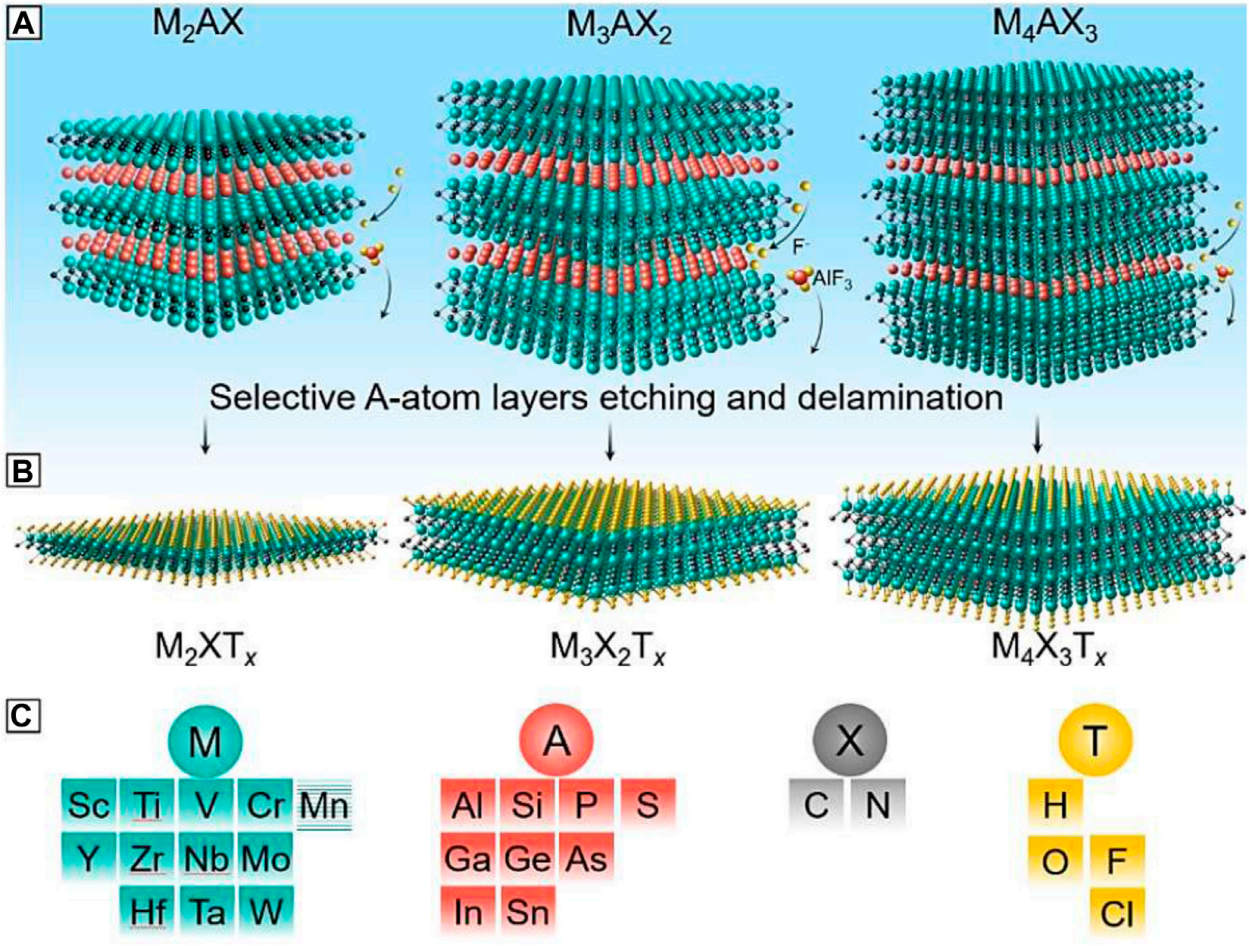
Different types of MXenes with different compositions with their precursor MAX phases. **(A)** MAX phases, **(B)** terminated MXenes after etching of MAX phases and **(C)** representation of M, A, X, and T. Adapted with the permission of (Hong et al., 2020)©Springer Nature 2020.

The different types of MXenes derived from their precursors through their selective etching processes are shown in Figure 1A, it showcases three categories of mono-M MAX phases: M_2_AX, M_3_AX_2_, and M_4_AX_3_, along with the selective etching procedure targeting the A-group layers (depicted by red atoms). In Figure 1B, it follows the selective etching process, MXenes emerge, characterized by the formation of surface terminations (depicted by yellow atoms) denoted as T. Figure 1C presents the potential elements for M, A, X, and T in both MAX and MXene phases. Additionally, although MXenes with orders as high as M_5_X_4_ have been successfully synthesized, they are not included in this table. It’s noteworthy that while Mn occupies the M sites in MAX phases, MXenes containing Mn have not been synthesized to date, indicated by the horizontally patterned background of Mn.

MXenes in addition to their exhibiting excellent electrical conductivity, mechanical strength, and chemical stability, often lack certain properties required for specific applications (Bilibana, 2023). By incorporating MXenes into different matrices such as polymers, ceramics, or metals, it can be exploited synergistic effects to achieve desired functionalities. For instance, MXene nanocomposites can offer improved mechanical strength, electrical conductivity, thermal stability, and even functionalities like electromagnetic interference shielding or energy storage (Gao et al., 2020). These nanocomposites find applications across various fields including energy storage devices like batteries and supercapacitors, electromagnetic shielding materials, sensors, catalysis, and biomedical devices. The versatility and tunability of MXene nanocomposites make them highly desirable for addressing specific needs in diverse technological domains (Perera et al., 2023). MXenes exhibit chemical stability, resisting oxidation and corrosion, contributing to the durability of nanocomposites in various environments (W. Wu et al., 2019a; M; Zhu et al., 2016b). MXenes can be synthesized with different transition metals, providing versatility in tailoring their properties for specific nanocomposite applications (Naguib et al., 2016).

In nanocomposite applications, MXenes are often combined with polymers, ceramics, or other materials to create hybrid materials with enhanced or multifunctional properties. The combination of these MXene properties allows for the development of advanced nanocomposites with diverse applications across various fields.

## 2 MXene, polymer, and their nanocomposites

MXene nanocomposites, combining the unique properties of MXenes with the versatility of polymers, offer enhanced mechanical, thermal, and electrical properties. These materials hold promise in applications such as flexible electronics and energy storage. Understanding fabrication techniques and interface engineering is crucial for tailoring MXene-polymer nanocomposites to specific needs. The MXene, Polymer, and their classification and nanocomposites are described briefly below.

### 2.1 MXenes

MXenes possess a distinctive set of properties that make them highly suitable for nanocomposite applications, particularly in the fields of biomedical science. MXenes have a unique 2D layered structure, providing a large surface area for interactions within nanocomposites. MXenes exhibit excellent electrical conductivity (Wang et al., 2019), making them advantageous for applications in electronics, sensors (Guo et al., 2019), and energy storage devices (George and Kandasubramanian, 2020). MXenes demonstrate impressive mechanical strength and stiffness, contributing to enhanced mechanical properties when incorporated into nanocomposites (Jimmy and Kandasubramanian, 2020). The surface of MXenes is rich in functional groups, such as hydroxyl and/or fluorine terminations, enabling easy surface modification and compatibility with other materials (Chen et al., 2020). MXenes typically possess a high specific surface area due to their layered structure, facilitating improved adsorption and reactivity in nanocomposite applications (Oyedotun et al., 2009). MXenes exhibit good thermal conductivity, making them suitable for applications in thermal management and devices requiring efficient heat dissipation. MXenes often demonstrate biocompatibility, a crucial property for their integration into nanocomposites for biomedical applications, such as drug delivery systems and medical implants. Some MXenes, like titanium carbide (Ti_3_C_2_T_x_), possess excellent photothermal properties, making them useful in nanocomposites for applications like cancer therapy (Z. Liu et al., 2018; C; Dai, Chen, et al., 2017; Lin et al., 2018; X; Han et al., 2018; C; Dai, Lin, et al., 2017). MXenes are available in various compositions (carbides, nitrides, or carbonitrides), allowing for versatility in tailoring nanocomposites for specific applications. MXenes display interesting optical properties, including strong light absorption, which can be harnessed for applications in sensing and optoelectronics (McKeen, 2017).

#### 2.1.1 Classification of MXenes on different basis

MXenes can be classified based on several different criteria, including composition, synthesis method, structural properties, and applications. The classification of MXenes based on various factors is listed in Table 1.

**TABLE 1:** Classification of MXenes on different basis.

Class. Basis	Types
Composition (Naguib et al., 2011)	Transitional metal: MXenes are primarily composed of transition metals such as titanium (Ti), vanadium (V), molybdenum (Mo), tungsten (W), niobium (Nb), etc.
	Element X: MXenes contain carbon (C), nitrogen (N), or both (carbonitrides) as the second element
Synthesis (Anasori, Lukatskaya, and Gogotsi, 2017)	Top-down: Involves etching of MAX phases (precursor materials) with strong acids or other chemical treatments to selectively remove the ‘A' layer, resulting in the formation of MXenes
	Bottom-up: Involves direct synthesis of MXenes from precursor materials or starting compounds, often through chemical vapor deposition (CVD) or other methods
Structure (Parajuli et al., 2022)	Layered: MXenes typically have a layered structure with transition metal layers sandwiched between ‘X' layers
	Surface functionalization: MXenes can be functionalized with various functional groups or ions on their surfaces, altering their properties and applications
Application (Lukatskaya et al., 2013; Lipatov et al., 2016; Shahzad et al., 2016)	Energy storage: MXenes are extensively used in energy storage devices such as batteries, supercapacitors, and fuel cells due to their high conductivity and large surface area
	Electronics: MXenes find applications in electronics, including transparent conductive films, flexible electronics, and electromagnetic interference shielding
	Catalysis: MXenes exhibit catalytic activity in various reactions, including hydrogen evolution, oxygen reduction, and water splitting
	Sensing: MXenes are utilized in gas sensing, biosensing, and electrochemical sensing applications due to their high surface area and conductivity
	Biomedical: Some MXenes show biocompatibility and are explored for drug delivery, photothermal therapy, tissue engineering, and bioimaging applications
Properties (Parajuli, Uppugalla, et al., 2023)	Electrical conductivity: MXenes possess high electrical conductivity, making them suitable for electronic and energy storage applications
	Mechanical properties: MXenes exhibit excellent mechanical strength and flexibility, which is advantageous for applications requiring robust materials
	Chemical stability: MXenes are chemically stable under various conditions, enhancing their suitability for catalytic and environmental applications
Layer thickness	MXenes can have varying layer thicknesses, ranging from a few atomic layers to thicker structures, influencing their properties and applications
Transitional Metals	Titanium: (Ti3C2, Ti2C, etc.): (Naguib et al., 2011)
	• Energy storage devices: Titanium-based MXenes are widely used in supercapacitors and lithium-ion batteries due to their high surface area, excellent electrical conductivity, and ability to intercalate ions
	• Electromagnetic interference (EMI) shielding: Their high electrical conductivity makes them suitable for shielding electromagnetic radiation in various electronic devices
	Vanadium (V2C, V2CTx, etc.): (Naguib et al., 2012)
	• Electrochemical sensors: Vanadium-based MXenes have shown promising applications in electrochemical sensing due to their high surface area and excellent electrical conductivity
	• Catalysis: They are explored as catalysts for various reactions, including hydrogen evolution reaction (HER), oxygen reduction reaction (ORR), and water splitting
	Molybdenum (Mo2CTx, Mo2TiC2Tx, etc.)(Halim, Kota, et al., 2016; Parajuli, Devendra, et al., 2021)
	• Lubrication: Molybdenum-based MXenes exhibit excellent lubricating properties, making them suitable additives in lubricant formulations for reducing friction and wear
	• Gas sensing: They have demonstrated sensitivity towards gases like ammonia and nitrogen dioxide, making them potential candidates for gas sensing applications
	Tungsten (W2CTx, W2TiC2Tx, etc.)(Koc and Kodambaka, 2000)
	• Photothermal therapy: Tungsten-based MXenes have been explored for their photothermal properties, which can be utilized in cancer therapy by converting light energy into heat to ablate cancer cells
	• Water purification: They have shown promise in removing heavy metal ions and organic pollutants from water due to their high adsorption capacity and selectivity
	Other (Nb2CTx, Mo2TiC2Tx, etc.)(Khazaei et al., 2019)
	• Flexible electronics: MXenes with suitable properties are being investigated for applications in flexible and transparent electronics, such as flexible electrodes and transparent conductive films
	• Biomedical applications: Some MXenes exhibit biocompatibility and are being explored for biomedical applications such as drug delivery, biosensing, and tissue engineering scaffolds

This classification scheme highlights the diverse nature of MXenes and their potential across multiple fields, from energy storage and electronics to catalysis, sensing, and biomedical applications depending also on the transitional metals.

### 2.2 Polymer

Polymers are large molecules composed of repeating structural units called monomers. These molecules are characterized by their long chains and versatile properties, making them essential in various industries and everyday products (Painter and Coleman, 2019). They can be natural, such as proteins and DNA, or synthetic, like plastics and rubbers. Synthetic polymers are typically created through polymerization processes, where monomers are chemically bonded together to form long chains or networks (McCrum, Buckley, and Bucknall, 1997). This process allows for the manipulation of properties like strength, flexibility, and durability, making polymers highly customizable for specific applications. Polymers play a vital role in modern society, being used in countless products ranging from packaging materials and textiles to medical devices and electronics. Their versatility, affordability, and ease of manufacturing have led to their widespread adoption across industries, driving innovation and technological advancement. However, concerns about environmental impact and sustainability have prompted efforts to develop biodegradable and eco-friendly alternatives to traditional polymers, reflecting the ongoing evolution of polymer science and engineering (Young and Lovell, 2008).

#### 2.2.1 Classification of polymers

Polymers can be classified into several types based on their structure, origin, properties and applications. Some common types of polymers are listed in Table 2 (Young and Lovell, 2008).

**TABLE 2:** Common types of polymers.

Classification	Types and properties
Structure (Figure 2)	1. Linear: They have long, straight chains of monomer units. Examples include polyethylene and polypropylene
	2. Branched: They have linear chains with occasional side branches stemming from the main chain. Examples include low-density polyethylene (LDPE) and certain types of polyethylene
	3. Cross-linked: They have covalent bonds between polymer chains, forming a network structure. This type of polymer exhibits high strength and rigidity. Examples include vulcanized rubber and epoxy resins
Origin	1. Natural: These polymers occur naturally and are often derived from renewable resources. e.g., proteins (silk and wool), cellulose, and natural rubber
	2. Synthetic: They are man-made and are typically derived from petrochemicals and extensively used in various industries due to their versatility and controllable properties. e.g. polyethylene, polypropylene, polyvinyl chloride (PVC), and polystyrene
Polymerization	1. Addition: They are formed by the repeated addition of monomer units, typically through a double or triple bond. Examples include polyethylene, polypropylene, and polyvinyl chloride (PVC)
	2. Condensation: They are formed by the condensation reaction between monomer units, often accompanied by the elimination of small molecules such as water or alcohol. Examples include nylon, polyester, and polycarbonate
Molecular forces	1. Thermoplastic: Thermoplastic polymers can be melted and reshaped multiple times without undergoing chemical degradation. They are held together by weak intermolecular forces. Examples include polyethylene, polypropylene, and polystyrene
	2. Thermosetting: They undergo irreversible chemical reactions during curing, forming a network structure that cannot be melted or reshaped. They exhibit high strength and dimensional stability at high temperatures. Examples include epoxy resins and phenolic resins
Applications (Painter and Coleman, 2019)	1. Packaging
	Polyethylene (PE): Widely used for packaging films, bags, and bottles due to its flexibility, toughness, and moisture resistance
	Polyethylene Terephthalate (PET): Commonly used for beverage bottles and food packaging due to its transparency, strength, and barrier properties
	Polypropylene (PP): Suitable for packaging containers, food containers, and flexible packaging due to its heat resistance, stiffness, and chemical resistance
	2. Construction Field
	Polyvinyl chloride (PVC): Utilized in pipes, window frames, flooring, and siding due to its durability, weather resistance, and versatility
	Polystyrene (PS): Used in insulation, packaging, and construction materials due to its lightweight nature and insulation properties
	Polyethylene (PE) and Polypropylene (PP): Employed in geomembranes, roofing membranes, and insulation materials due to their waterproofing and durability
	3. Automotive field
	Polyurethane (PU): Used in car seats, foam cushions, and interior trim due to its comfort, durability, and vibration-dampening properties
	Acrylonitrile Butadiene Styrene (ABS): Utilized in automotive interior and exterior parts, such as dashboard components and body panels, due to its impact resistance and toughness
	Polyethylene (PE) and Polypropylene (PP): Employed in automotive bumpers, fuel tanks, and interior components due to their lightweight nature and impact resistance
	4. Medical field
	Polymethyl Methacrylate (PMMA): Used in medical devices such as intraocular lenses, bone cement, and dental materials due to its optical clarity and biocompatibility
	Polyethylene (PE) and Polypropylene (PP): Utilized in medical packaging, syringes, and implants due to their inertness, stabilizability, and biocompatibility
	Polyvinyl Chloride (PVC): Employed in medical tubing, blood bags, and IV containers due to its flexibility, transparency, and ease of sterilization
	5. Electronics field
	Polyethylene Terephthalate (PET): Used in electronic housings, insulating films, and printed circuit boards due to its dimensional stability and electrical insulation properties
	Polyamides (PI): Utilized in flexible circuits, insulating coatings, and electronic components due to their high-temperature stability, chemical resistance, and electrical insulation properties
	Polyethylene (PE) and Polypropylene (PP): Employed in battery casings, cable insulation, and electronic packaging due to their electrical insulation, thermal stability, and chemical resistance
Conducting polymer (Kurra, Jiang, and Alshareef, 2015)	Polypyrrole (PPy): It is one of the most extensively studied conducting polymers. It exhibits high conductivity, good environmental stability, and processability. Polypyrrole can be synthesized via chemical oxidation of pyrrole monomers, forming a highly conjugated polymer chain
	Polyaniline (PANI): It is another widely researched conducting polymer. It can be synthesized in various oxidation states, each exhibiting different electrical and optical properties. Polyaniline is relatively easy to process and shows good environmental stability, making it suitable for various applications such as sensors, actuators, and energy storage devices
	Polythiophene (PTh): Polythiophene and its derivatives are conducting polymers with high stability and good processability. They can be synthesized with different side chains and substituents to tailor their properties for specific applications. Polythiophenes are commonly used in organic electronic devices, including organic photovoltaics and organic light-emitting diodes (OLEDs)
	Polyacetylene (PA): It was one of the first conducting polymers to be discovered and studied. It exhibits high conductivity when doped with suitable dopants, but it is highly sensitive to oxygen and moisture, limiting its practical applications. However, polyacetylene has paved the way for the development of other conducting polymers with improved stability and performance
	Poly (3, 4-ethylenedioxythiophene (PEDOT): It is a conducting polymer known for its high conductivity, transparency, and flexibility. It is commonly used as a transparent electrode in organic electronics, touchscreens, and electrochromic devices. PEDOT can be synthesized through various methods, including chemical oxidation and electrochemical polymerization

**FIGURE 2:**
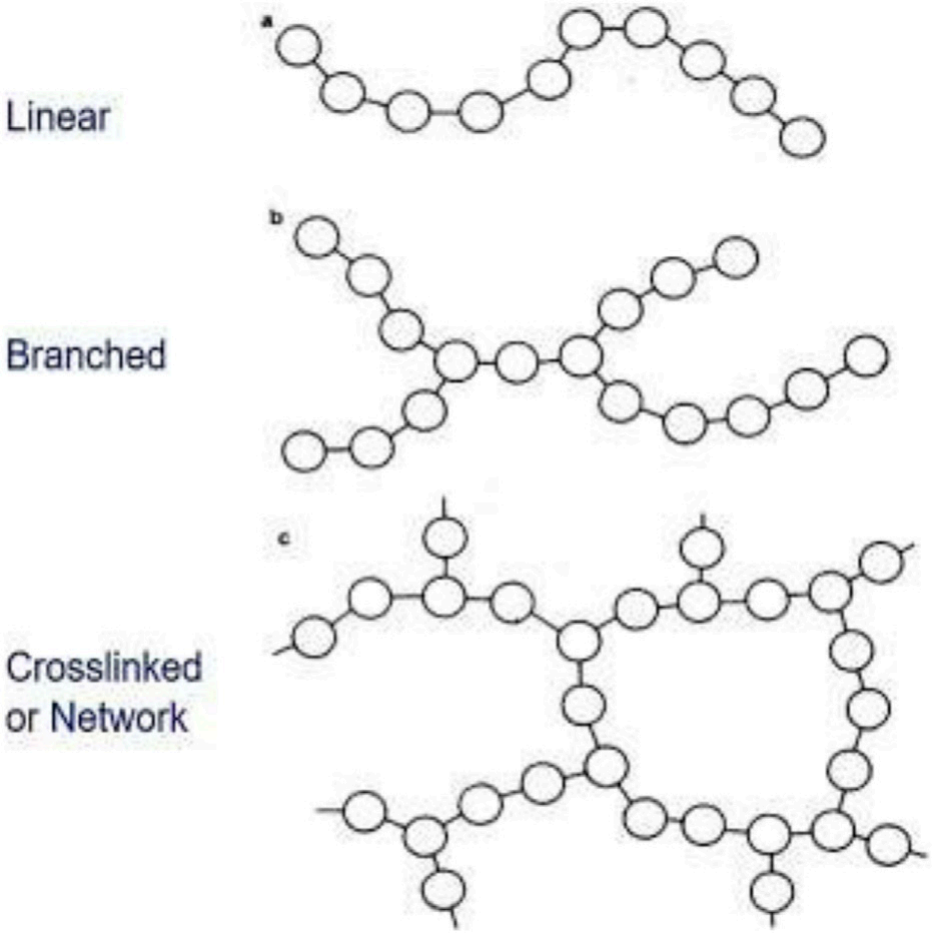
Types of Polymers: **(A)** linear, **(B)** branched, and **(C)** cross-linked or network.

### 2.3 MXene nanocomposites

MXene nanocomposites are a class of materials that have garnered significant attention due to their unique properties and potential applications. (Parajuli et al., 2022). MXene nanocomposites are formed by incorporating MXene nanosheets into a matrix material, such as polymers, ceramics, or metals, to enhance or impart specific properties. The addition of MXenes can improve mechanical strength, electrical conductivity, and thermal stability of the composite material. Research in MXene nanocomposites has demonstrated their potential in various fields (Aghamohammadi, Amousa, and Eslami-Farsani, 2021). For instance, MXene/polymer nanocomposites have shown promise in flexible electronics and sensors due to their excellent electrical conductivity and mechanical flexibility. In energy storage applications, MXene-based nanocomposites have been explored for supercapacitors and batteries, where they offer high specific capacitance and stability. One example of a study in MXene nanocomposites is the work by Liu et al. (2021), where they developed MXene/polymer nanocomposites for electromagnetic interference shielding applications. The study demonstrated that the incorporation of MXene nanosheets into the polymer matrix significantly enhanced the electromagnetic interference shielding effectiveness of the composite material (Zhang and Gu, 2022).

### 2.4 Polymer nanocomposites

Polymer nanocomposites are a class of materials composed of a polymer matrix reinforced with nanoscale fillers or additives. These nanofillers typically have at least one dimension in the nanometer range, providing unique properties and enhanced performance compared to traditional composite materials (Koo, 2019). The incorporation of nanofillers into polymers can result in significant improvements in mechanical, thermal, electrical, and barrier properties (Pinto et al., 2023). Common types of nanofillers used in polymer nanocomposites include nanoparticles such as metal oxides (e.g., silica, alumina), carbon-based materials (e.g., carbon nanotubes, graphene), and clay minerals (e.g., montmorillonite).

Polymer nanocomposites offer numerous advantages over conventional composites, including:

• Enhanced mechanical properties such as increased stiffness, strength, and toughness.
• Improved thermal stability and flame retardancy due to the barrier effect of nanofillers.
• Enhanced electrical conductivity or dielectric properties, making them suitable for electronic and electrical applications.
• Increased gas barrier properties, making them ideal for packaging materials.
• Reduced weight and improved fuel efficiency in transportation applications.

The fabrication of polymer nanocomposites involves various techniques such as melt blending, solution mixing, *in situ* polymerization, and electrospinning. The selection of nanofillers, their dispersion within the polymer matrix, and the processing conditions play crucial roles in determining the properties and performance of the nanocomposite material. Polymer nanocomposites find applications in diverse industries, including automotive, aerospace, electronics, packaging, biomedical, and energy storage. Ongoing research in this field aims to further optimize the properties of nanocomposites and explore novel applications, paving the way for the development of advanced materials with tailored properties and multifunctionality.

### 2.5 MXene-polymer nanocomposites

By integrating MXenes, renowned for their exceptional conductivity and mechanical properties, into polymer matrices, the resulting nanocomposites exhibit enhanced functionalities such as improved electrical conductivity, mechanical strength, and thermal stability (Gong et al., 2021). These tailored properties make MXene polymer nanocomposites highly promising for a wide array of applications, including flexible electronics, energy storage devices like batteries and supercapacitors, electromagnetic interference shielding materials, and even biomedical applications (Bilibana, 2023). Moreover, the versatility of polymers allows for easy processing and shaping, further expanding the potential applications and manufacturability of MXene polymer nanocomposites. The synthesis of MXene polymer nanocomposites represents a synergistic approach to harnessing the strengths of both materials, paving the way for advancements in numerous fields (Riazi et al., 2021).

From few years back, we have studied the MXenes and Ferrites (Parajuli, Raghavendra, et al., 2021; Parajuli, Murali, Rao, Ramakrishna, et al., 2022; Parajuli, Taddesse, Murali, and Samatha 2022; Parajuli, Murali, Raghavendra, et al., 2023; Parajuli, Murali, Samatha, et al., 2023) separately and their mixtures with the most exploited material in the society polymer in the form of their nanocomposites (Parajuli et al., 2022; Parajuli, Uppugalla, et al., 2023). We found them most efficient combination among the recently developed other nanocomposites which are far efficient than their pristine form.

Nanocomposites represent a cutting-edge technology with transformative potential in biomedical fields. Their ability to address specific challenges and provide tailored solutions underscores their significance in advancing healthcare and promoting environmental sustainability. The need for advanced materials like MXenes-polymer nanocomposites stems from their exceptional properties, versatility, and potential to address challenges in diverse applications, ranging from healthcare protection to energy storage and beyond. Their unique combination of attributes positions them as key players in advancing technological solutions for the future. In this article, the synthesis of MXene Polymer nanocomposites, their biomedical and environmental aspects along with the issues, challenges, and perspectives are discussed. MXenes properties for nanocomposite applications.

### 2.6 MXenes and polymers compatibility for nanocomposite

The compatibility of MXene with polymers is a crucial aspect in the formation of nanocomposites, determining the effectiveness and performance of the resulting hybrid materials (X. Chen et al., 2020). The compatibilities of MXene with polymers for nanocomposite formation are:

#### 2.6.1 Surface functional groups

MXenes possess surface functional groups, such as hydroxyl (-OH) or fluorine (-F), which enhance their compatibility with polymers. These functional groups provide active sites for bonding and interactions with polymer chains.

#### 2.6.2 Chemical affinity

The surface chemistry of MXene allows for strong interactions with various polymers. The presence of transition metal terminations and surface functional groups promotes chemical compatibility, ensuring a stable interface between MXene and polymer components.

#### 2.6.3 Electrostatic interactions

The charged nature of MXene surfaces facilitates electrostatic interactions with polymers, especially those with opposite charges. This can contribute to improved dispersion and stability of MXene within the polymer matrix.

#### 2.6.4 Enhanced mechanical properties

The mechanical strength of MXene, coupled with its compatibility with polymer matrices, results in nanocomposites with enhanced mechanical properties. This is particularly beneficial in applications requiring increased strength and durability.

#### 2.6.5 Synergistic effects

The combination of MXene and polymers often leads to synergistic effects, where the unique properties of each component complement and enhance the overall performance of the nanocomposite. This synergy can result in improved conductivity, mechanical strength, or other desired characteristics.

#### 2.6.6 Versatility of polymer selection

MXenes can be compatible with a wide range of polymers, including but not limited to, thermoplastics, thermosets, and elastomers. This versatility allows for the tailoring of nanocomposite properties to suit specific applications.

#### 2.6.7 Improved processability

MXene’s compatibility with polymers facilitates the processing and fabrication of nanocomposites. Techniques such as solution mixing, melt blending, or *in situ* polymerization can be employed to achieve homogeneous dispersion and integration.

#### 2.6.8 Biocompatibility

MXenes can be engineered to be biocompatible, making them suitable for integration into biopolymer matrices. This opens up opportunities for applications in biomedical nanocomposites, such as drug delivery systems and tissue engineering (K. Chen et al., 2017).

The compatibility of MXene with polymers is a critical factor in the successful formation of nanocomposites. The chemical affinity, surface functional groups, and synergistic effects contribute to creating hybrid materials with tailored properties, expanding the range of applications in various fields.

## 3 Synthesis of MXene-polymer nanocomposites

**FIGURE 3:**
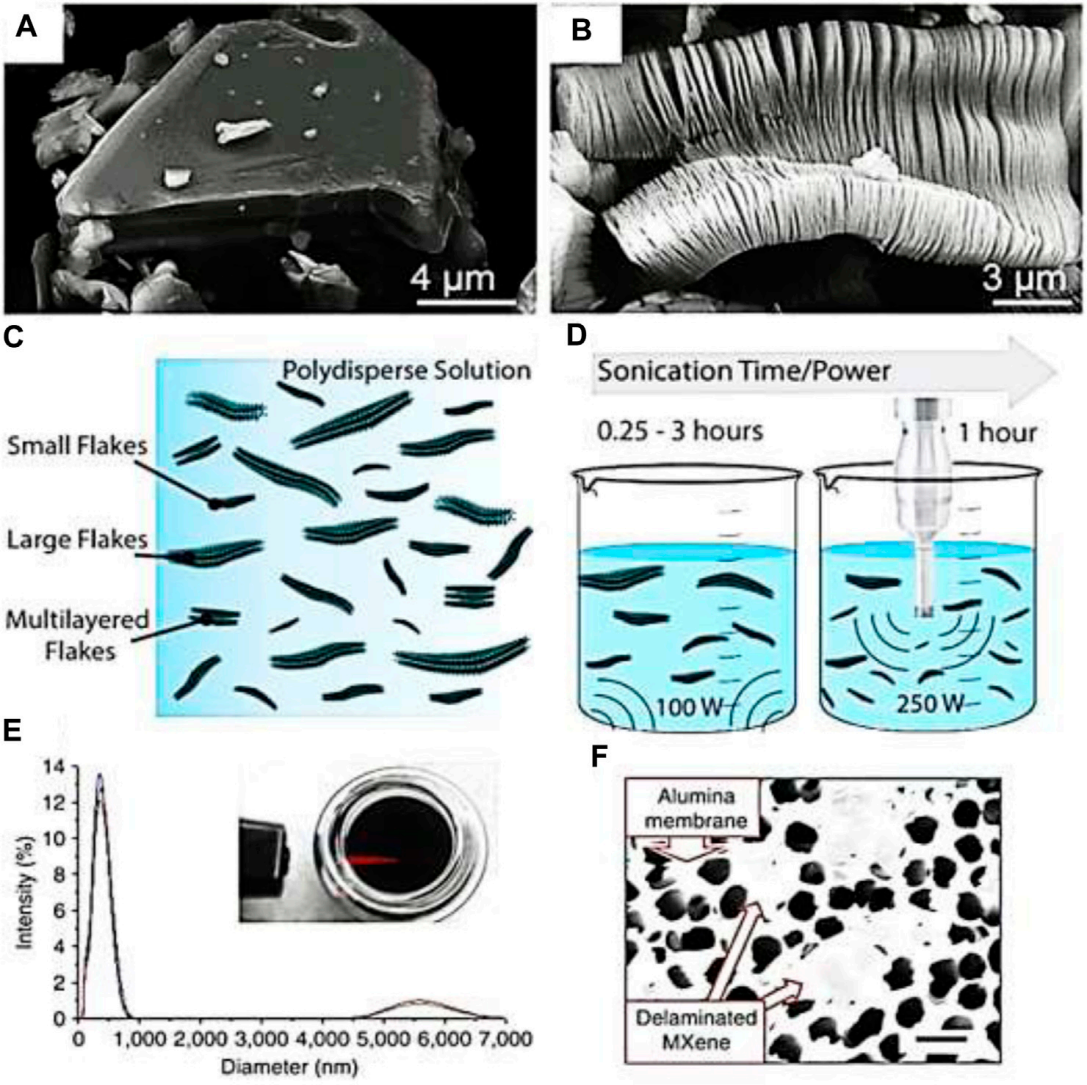
**(A)** Scanning electron microscopy (SEM) depiction of Ti_3_AlC_2_ particle **(B)** Formation of Ti_3_C_2_T_z_ after etching in hydrofluoric acid (HF). **(C)** Diagrammatic representation illustrating MXene suspension exhibiting various flake sizes. **(D)** Representation demonstrating the impact of sonication on dimensions of MXene flakes, with increasing sonication time and power. **(E)** Light scattering analysis of a typical colloidal MXene suspension post-sonication, accompanied by an inset image demonstrating the Tyndall effect. **(F)** SEM micrograph displaying drop-cast colloidal MXene on a porous alumina substrate, showcasing transparent delaminated MXene sheets. **(A, B)** are reproduced from Naguib et al., 2012. **(C, D)** are reproduced from Maleski et al., 2018. **(E, F)** are reproduced from Mashtalir et al., 2013.

Synthesizing MXene-polymer nanocomposites involves various techniques, each with its advantages, limitations, and recent advancements. In most cases, the MXenes are synthesized from etching processes. The layered structures in colloidal solution form obtained from etching can be sonicated and delaminated to get a few layered MXenes (Carey and Barsoum, 2021) as shown in Figure 3. Their synthesis involves integrating MXene nanosheets into a polymer matrix to create materials with enhanced properties. The detail of the synthesis of MXene-Polymer nanocomposites is found in our recently published articles (Parajuli et al., 2022; Parajuli, Uppugalla, et al., 2023).

### 3.1 Steps of synthesis methods

The synthesis typically involves the following steps.

1 Preparation of MXene Nanosheets: MXene nanosheets are synthesized through methods such as chemical etching of MAX phases or exfoliation of precursor materials. The resulting MXene nanosheets are typically functionalized to improve their dispersion and compatibility with the polymer matrix.
2 Preparation of Polymer Matrix: The polymer matrix is prepared separately, often by dissolving the polymer in a suitable solvent to form a polymer solution. The choice of polymer depends on the desired properties of the nanocomposite and the intended application.
3 Dispersion of MXene in Polymer solution: The MXene nanosheets are then dispersed in the polymer solution using techniques such as sonication or mechanical stirring. Proper dispersion is crucial to ensure uniform distribution of MXene within the polymer matrix and to prevent aggregation.
4 Fabrication of Nanocomposite: The dispersed MXene-polymer solution is then subjected to a suitable fabrication method to form the nanocomposite material. Common techniques include solution casting, spin-coating, or electrospinning, depending on the desired morphology and application of the nanocomposite.
5 Characterization and Optimization: The synthesized MXene-polymer nanocomposite is characterized to evaluate its structural, mechanical, electrical, and thermal properties. Optimization of synthesis parameters may be performed to achieve desired properties such as improved mechanical strength, conductivity, or thermal stability.
6 Application Testing: The MXene-polymer nanocomposite is then evaluated for its performance in specific applications such as energy storage, electromagnetic interference shielding, sensors, or biomedical devices. The nanocomposite’s properties are tailored to meet the requirements of the intended application.

The synthesis of MXene-polymer nanocomposites involves careful control of MXene dispersion, polymer compatibility, and fabrication techniques to achieve nanocomposites with enhanced properties suitable for various practical applications.

### 3.2 Types of synthesis methods

#### 3.2.1 Selective etching

The synthesis of MXenes and hence the MXene Nanocomposites, involves a multistep process, primarily starting with the selective etching of layers from a precursor material known as a MAX phase. The etching of MXene typically refers to a process of selectively removing the “A” layer (typically aluminum) from the MXene structure, leaving behind a porous 2D material with various applications. The etching process is essential because it alters the surface chemistry and structure of MXene, allowing for tuning of its properties and enabling various applications. The etching of MXene can be used to control the surface chemistry, surface area, and interlayer spacing, which are crucial for applications such as energy storage devices (like batteries and supercapacitors), electromagnetic interference shielding, catalysis, sensors, and more. The steps for the synthesis of MXene Polymer nanocomposite by selective etching process are as below.

##### 3.2.1.1 Selective etching

The primary step in MXene synthesis involves the selective etching of the A-element layers from the MAX phase. Common etchants include hydrofluoric acid (HF) or a combination of hydrochloric acid (HCl) and an oxidizing agent. The etching process exposes the MXene layers, creating a 2D structure (Naguib et al., 2012).

##### 3.2.1.2 Washing and delamination

After etching, the resulting MXene flakes are thoroughly washed to remove residual etchants and by-products. This step is crucial for the removal of any remaining A-element residues. Delamination processes, such as intercalation of ions or molecules, may be employed to exfoliate MXene layers and enhance their dispersibility (Ling et al., 2014).

##### 3.2.1.3 Surface functionalization

MXene surfaces often have terminations, such as hydroxyl (-OH) or fluorine (-F), which affect their properties. Surface functionalization can be performed to introduce desired functional groups, enhancing their compatibility with other materials or improving specific characteristics.

MXenes are frequently integrated into polymer matrices to form nanocomposites. This can be achieved through methods like solution mixing, melt blending, or *in situ* polymerization. The choice of polymer and the synthesis method can be tailored to achieve the desired properties for specific applications (Khazaei et al., 2013; Ashton et al., 2016; T; Hu et al., 2015).

##### 3.2.1.4 Drying and powder formation

The resulting MXene dispersion is often dried to produce a powder form for ease of handling and storage. Techniques like freeze-drying or spray-drying are commonly employed to preserve the 2D structure.

##### 3.2.1.5 MXene-polymer nanocomposite formation

**FIGURE 4:**
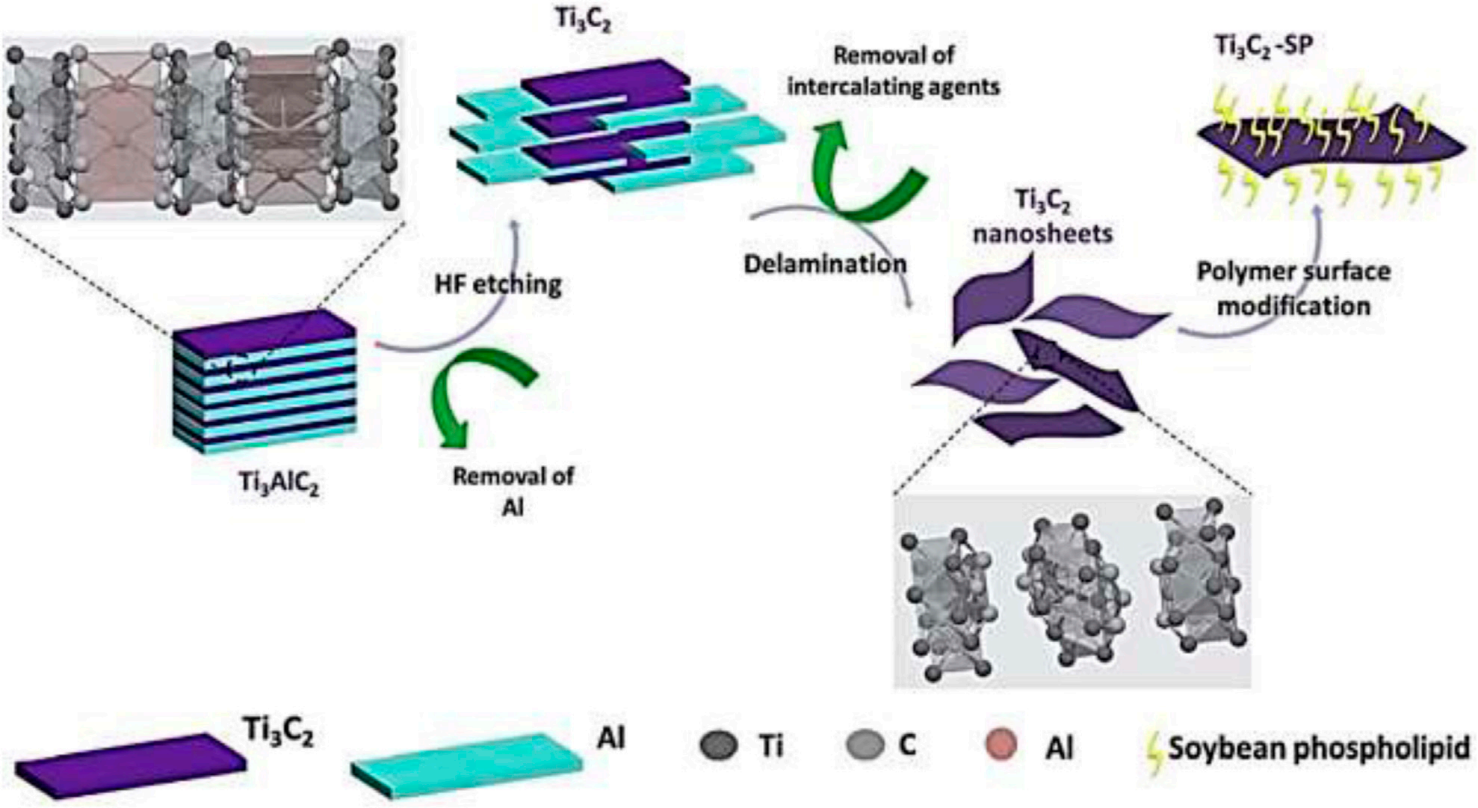
Synthesis cycle of Ti_3_C_2_-SP nanocomposite (Jain et al., 2013).

The schematic representation of the synthesis cycle for the MXene-SP composite is illustrated in Figure 4. The bottom-up methodology can also be employed for MXene synthesis through techniques such as EVD (Manawi et al., 2018).

**FIGURE 5:**
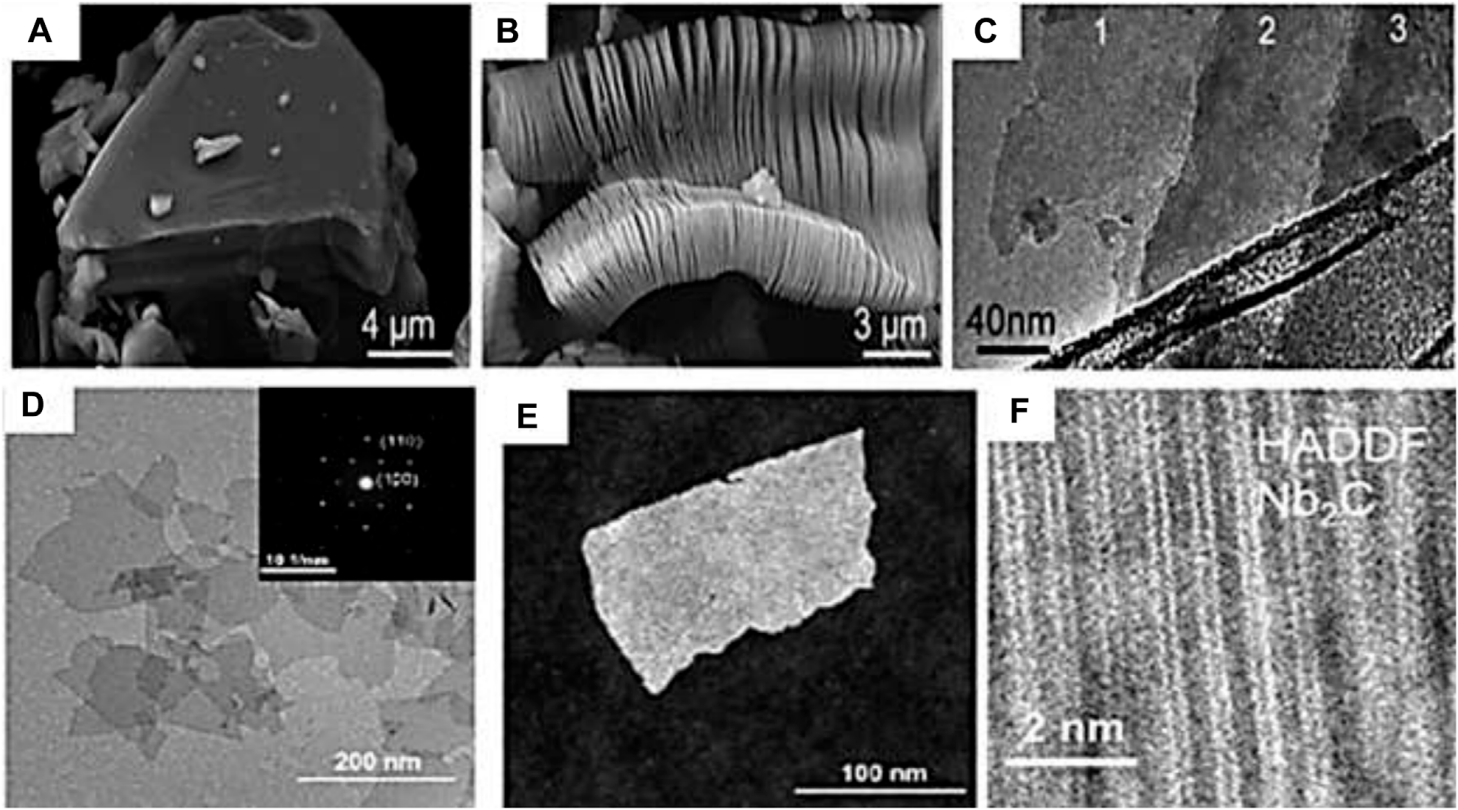
**(A)** SEM micrographs of Ti_3_AlC_2_ before and **(B)** after etching. **(C)** TEM images of Ti_3_C_2_ after etching (Naguib et al., 2012) © American Chemical Society (2012). **(D, E)** TEM images (Bright and dark field Nb_2_C). **(F)** High resolution HAADF-STEM image. ^©^ American Chemical Society (2019).

Figure 5 presents scanning transmission electron microscopy (STEM) images of Ti_3_AlC_2_ before and after etching, high-resolution transmission electron microscope (HRTEM) of Ti_3_C_2_ after etching, bright and dark selected area electron diffraction (SAED) of Nb_2_C, high angular annular dark field (HAADF) respectively, and showcasing hexagonally layered intercalated nanosheets with a space group of P63/mmc and atomic arrangement dependent on “n,” with lateral dimensions ranging from 0.5 to 200 nm and a thickness of a few nanometers (Naguib et al., 2014) (X. Ren et al., 2019).

#### 3.2.2 Solution mixing

They are simple and cost-effective, suitable for a wide range of polymers, and allow for precise control over the composition. However, they are limited to polymers soluble in the chosen solvent and may require additional steps for complete mixing and dispersion. Advanced mixing techniques, such as sonication or high-shear mixing, can be used to improve dispersion. Solution mixing is a common and straightforward method for preparing MXene-polymer nanocomposites (Maleski, Mochalin, and Gogotsi, 2017).

##### 3.2.2.1 Material and method

The MXene of interest, such as titanium carbide (Ti_3_C_2_T_x_) or others, is prepared through appropriate synthesis methods. The chosen polymer, compatible with the solvent is used for MXene dispersion. Common polymers include polyvinyl alcohol (PVA), polyethylene glycol (PEG), or others. A solvent in which both MXene and the polymer are soluble. Common solvents include water, N-methyl-2-pyrrolidone (NMP), or dimethyl sulfoxide (DMSO). A sonicator is needed to assist in dispersing MXene and polymer in the solvent. For continuous mixing during the preparation is done by magnetic stirrer or stirring rod. A suitable container is used for mixing and storing the nanocomposite solution.

The MXene is dispersed in the chosen solvent, with uniform dispersion often achieved through sonication. For example, a desired amount of MXene powder is added to the solvent, and the mixture is sonicated for a specific duration until a well-dispersed MXene solution is obtained. The polymer is dissolved in the same solvent used for MXene dispersion, with the solution stirred until the polymer is fully dissolved, forming a clear polymer solution. The MXene solution is slowly added into the polymer solution while continuously stirring, with this process carried out dropwise to ensure a gradual and homogeneous mixture. The combined solution is stirred for an extended period to ensure proper mixing and distribution of MXene within the polymer matrix. Depending on the desired concentration of MXene in the nanocomposite, the ratio of MXene to polymer solutions is adjusted, with this step potentially requiring optimization based on the specific application. The resulting nanocomposite solution is characterized to ensure the desired properties, with techniques such as UV-Vis spectroscopy, Fourier-transform infrared spectroscopy (FTIR), or other relevant methods employed. Depending on the application, the nanocomposite solution can be cast into films or coatings or dried to obtain the final nanocomposite material.

##### 3.2.2.2 Considerations

In this process, some special considerations are needed. The concentration of MXene, polymer, and the ratio between them should be optimized based on the desired properties of the nanocomposite. The choice of solvent is critical, as it affects the solubility of both MXene and the polymer. Consideration should be given to environmental and safety aspects when selecting solvents. Proper sonication is essential to ensure a well-dispersed MXene solution, preventing agglomeration. Sufficient stirring time is crucial for achieving a homogeneous nanocomposite solution.

#### 3.2.3 *In-situ* polymerization

They are a direct synthesis of nanocomposites during polymerization and have good control over the nanocomposite structure. They are suitable for a variety of polymer matrices. However, they are limited to polymers that can undergo *in situ* polymerization, and reaction conditions may affect MXene properties. The development of controlled polymerization methods for better control over nanocomposite structure. *In situ* polymerization is a method for preparing MXene-polymer nanocomposites in which the polymerization of monomers occurs directly in the presence of dispersed MXene sheets.

**FIGURE 6:**
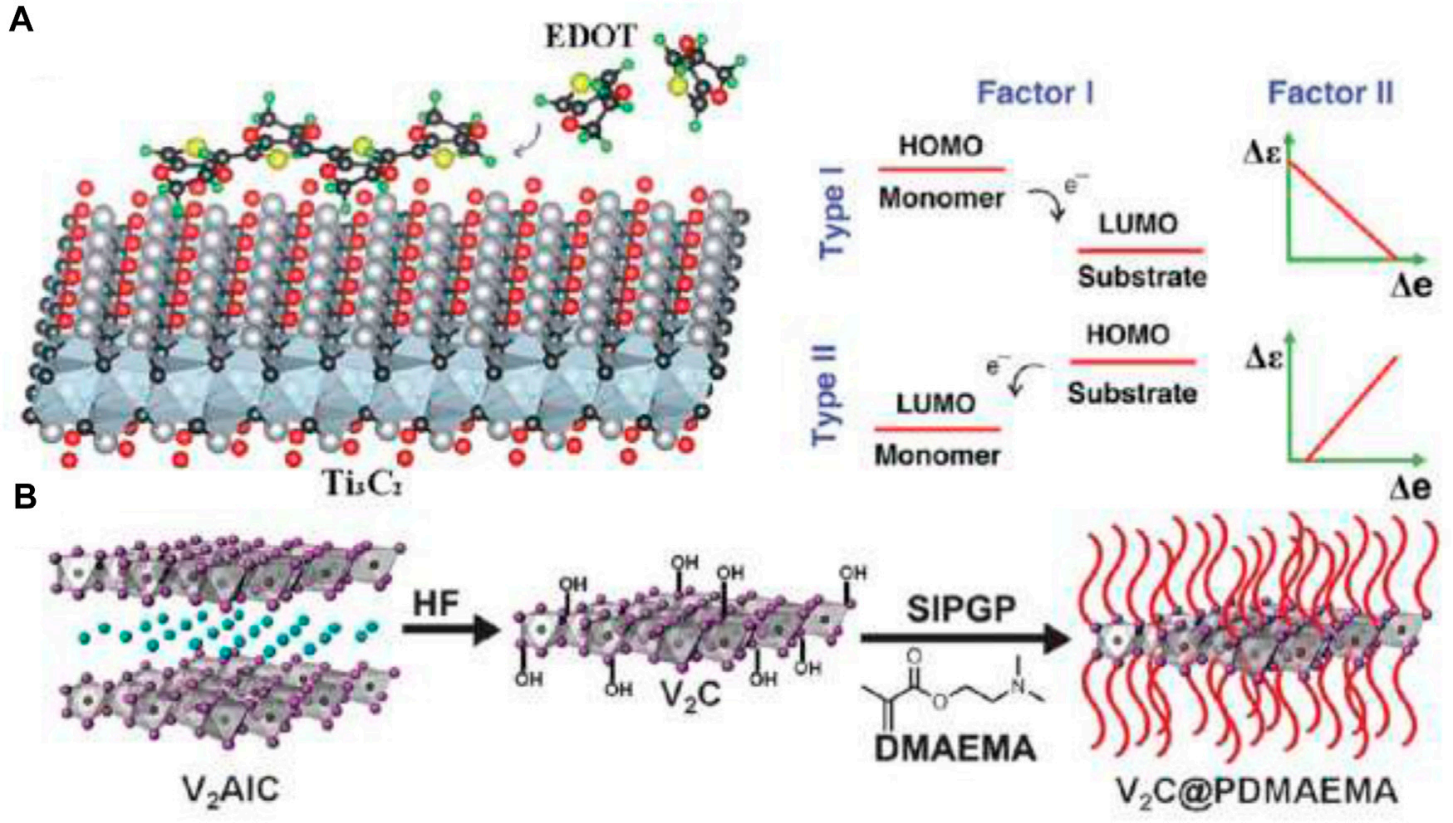
Synthesis of MXene/polymer composites through *in situ* polymerization: **(A)** Schematic depiction of the polymerization of EDOT on the surface of MXene and the process of charge transfer during polymerization, adapted from (C. Chen et al., 2017) ©2017 Royal Society of Chemistry and **(B)** Schematic illustration of the preparation of V2C@PDMAEMA composite. **(A)**: Adapted with permission from reference (J. Chen et al., 2014) ©2015 Royal Society of Chemistry.

Due to limited polymerization energy, only certain types of monomers can undergo polymerization on the surface of MXenes through methods such as physical agitation, UV radiation, or electrochemical processes (C. Chen et al., 2017) (J. Chen et al., 2014; Boota et al., 2016). Chen et al. proposed two primary electron transfer mechanisms during the charge-transfer-induced polymerization of 3, 4-ethylenedioxythiophene (EDOT) under agitation. In one mechanism, electrons transition from the high-energy orbits (HOMOs) of the monomer to the lower-energy orbits (LUMOs) of the MXene, while in the other, electrons shift from the HOMOs of the MXene to the LUMOs of the monomer (Figure 6A) (C. Chen et al., 2017). Additionally, proximity between the monomer and MXene surface is crucial for efficient electron transfer during polymerization. Leveraging hydroxyl and fluorine terminations, 2-(dimethylamino) ethyl methacrylate (DMAEMA) was successfully polymerized on the surface of V_2_C (Figure 6B) (J. Chen et al., 2014), leading to the formation of PDMAEMA grafted V_2_C with demonstrated sensitivity to CO2 and temperature. Furthermore, MXene/polypyrrole (PPy) composites were synthesized via electrochemical polymerization by Zhu et al., involving the electrolysis of a pyrrole-containing solution with a Ti_3_C_2_-modified electrode. Although *in situ* polymerization ensures a uniform distribution of polymers on the MXene surface, it may alter the properties of MXene materials during the process.

##### 3.2.3.1 Material and method

In this method, the monomers are required for polymerization to make nanocomposite with the MXene of interest, such as titanium carbide (Ti_3_C_2_T_x_). Common monomers include acrylic acid, acrylamide, or other suitable monomers compatible with the polymerization conditions. A suitable initiator to initiate the polymerization reaction. Common initiators include ammonium persulfate (APS), azobisisobutyronitrile (AIBN), or others, depending on the polymerization mechanism. A solvent in which both MXene and the monomers are dispersible. Common solvents include water, N-methyl-2-pyrrolidone (NMP), or dimethyl sulfoxide (DMSO). A suitable reaction vessel equipped with a stirring mechanism and temperature control is needed.

The MXene is dispersed in the chosen solvent to achieve a stable MXene dispersion, potentially involving sonication for uniform dispersion. The desired monomers are dissolved in the solvent to prepare the monomer solution, with the concentration adjusted based on the nanocomposite (NC) properties. e.g., epoxies (Sliozberg et al., 2020; H; Zhang et al., 2016; Zou et al., 2018) and polydimethylsiloxane (PDMS) like polymers are mixed from one side.

The most common reports of *insitu* polymerizations for MXene polymer NCs are of curing systems, such as epoxies (Hatter et al., 2020; Sliozberg et al., 2020) (A. Feng et al., 2020) and PDMS (D. Hu et al., 2020) (X. Wu et al., 2020)(D. Wang et al., 2020) aqueous solutions have been reported in the case of PPy (Zhu et al., 2016a; Boota et al., 2016; Zhang et al., 2020), polyaniline (Vahidmohammadi et al., 2018; Y; Ren et al., 2018; Wei et al., 2019; Vahidmohammadi et al., 2018; Xu et al., 2020), PAM(Zhang et al., 2019), PAM/PVA (Liao et al., 2019) and PEDOT (Chen et al., 2017; Qin et al., 2019), that can be polymerized with acetonitrile (Li et al., 2019).

The initiator is added to the monomer solution, its amount is determined by polymerization kinetics and reaction rate preferences. The MXene dispersion is slowly added into the monomer solution while continuously stirring to uniformly disperse MXene sheets. Polymerization is initiated by raising the temperature or other suitable methods activating the initiator. The polymerization reaction proceeds for the specified duration under controlled conditions. The resulting MXene-polymer nanocomposite is characterized using techniques such as FTIR, SEM, or other relevant methods. The nanocomposite material is isolated from the reaction mixture through filtration or other separation techniques and washed to remove unreacted monomers or by-products. Finally, the nanocomposite material is dried to obtain the final product.

##### 3.2.3.2 Considerations

The polymerization conditions, including temperature, initiator concentration, and reaction time, to achieve the desired properties of the nanocomposite are optimized. Continuous stirring are ensured during the polymerization reaction to promote uniform dispersion of MXene sheets and facilitate homogeneous polymerization. Thorough characterization of the nanocomposite material are done to evaluate its structure, morphology, and properties.

*In-situ* polymerization offers precise control over the nanocomposite structure and properties, making it a valuable method for preparing MXene-polymer nanocomposites with tailored characteristics for specific applications. Adjustments can be made to this general procedure based on the choice of monomers, initiators, or MXene types.

#### 3.2.4 Melt blending

**FIGURE 7:**
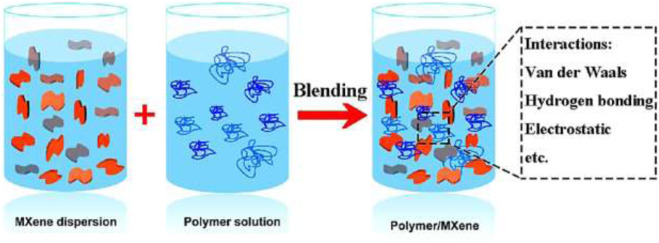
Creation of MXene/polymer composites through *ex-situ* solution blending. Reproduced with authorization from reference (Mirkhani et al., 2019). © 2019 American Chemical Society.

Melt blending is a commonly used method for preparing MXene-polymer nanocomposites, particularly when working with thermoplastic polymers (Figure 7). They are scalable and applicable to various polymer matrices, suitable for industrial-scale production, and are simple with cost-effective. However, they are limited to thermoplastic polymers and may require high processing temperatures. The optimization of processing parameters improves dispersion and mechanical properties (Sheng et al., 2019).

*Ex-situ* blending methods (Figure 7) are frequently utilized due to their ease in regulating the properties of polymer and MXene precursors. Leveraging their relatively modest interactions, such as van der Waals attraction, hydrogen bonding force, and electrostatic interaction, MXene/polymer composites are predominantly produced through solution mixing (Figure 7).

##### 3.2.4.1 Material and method

The MXene of interest, such as titanium carbide (Ti_3_C_2_T_x_) or others, is prepared through appropriate synthesis methods. The chosen thermoplastic polymer, is compatible with the processing temperature and MXene dispersion. A twin-screw extruder or internal mixer capable of high-temperature processing and efficient mixing. Additives such as compatibilizers, plasticizers, or stabilizers may be used to improve dispersion or enhance properties.

The MXene powder is properly dried to remove any residual moisture, as moisture can affect processing and properties. The thermoplastic polymer pellets are pre-dried to remove moisture and ensure uniform processing, with any desired additives optionally incorporated into the polymer pellets. The dried MXene powder and polymer pellets are loaded into the mixing equipment in the desired ratios, with the loading ratio adjusted based on the desired properties of the nanocomposite. The mixing equipment is heated to the appropriate processing temperature, typically above the melting point of the polymer. The mixing process is started, allowing the MXene powder and polymer pellets to melt and blend thoroughly, with high shear forces generated during mixing helping to disperse MXene within the polymer matrix. After thorough mixing, the molten mixture is extruded or discharged from the mixing equipment onto a cooling conveyor or casting surface. The mixture is allowed to cool and solidify, forming the MXene-polymer nanocomposite in the desired shape (e.g., pellets, sheets, or films). Further processing steps such as pelletizing, compression molding, or injection molding may be applied to the resulting nanocomposite material depending on the intended application. The nanocomposite material is characterized to evaluate its structure, morphology, and properties, with techniques such as SEM, XRD, or mechanical testing employed.

##### 3.2.4.2 Consideration

The processing temperature should be carefully controlled to ensure proper melting of the polymer and dispersion of MXene while avoiding degradation of either component. Sufficient mixing time is essential to achieve uniform dispersion of MXene within the polymer matrix. Longer mixing times may be required for higher loading levels or less compatible polymer-MXene combinations. The incorporation of additives, such as compatibilizers or plasticizers, may improve the dispersion and compatibility of MXene within the polymer matrix. Melt blending involves high temperatures and mechanical shear forces. Proper safety precautions should be followed to prevent accidents or injuries.

Melt blending offers scalability and versatility, making it suitable for industrial-scale production of MXene-polymer nanocomposites. Adjustments to processing parameters can be made based on the specific polymer-MXene combination and intended application requirements.

#### 3.2.5 Layer-by-layer assembly

**FIGURE 8:**
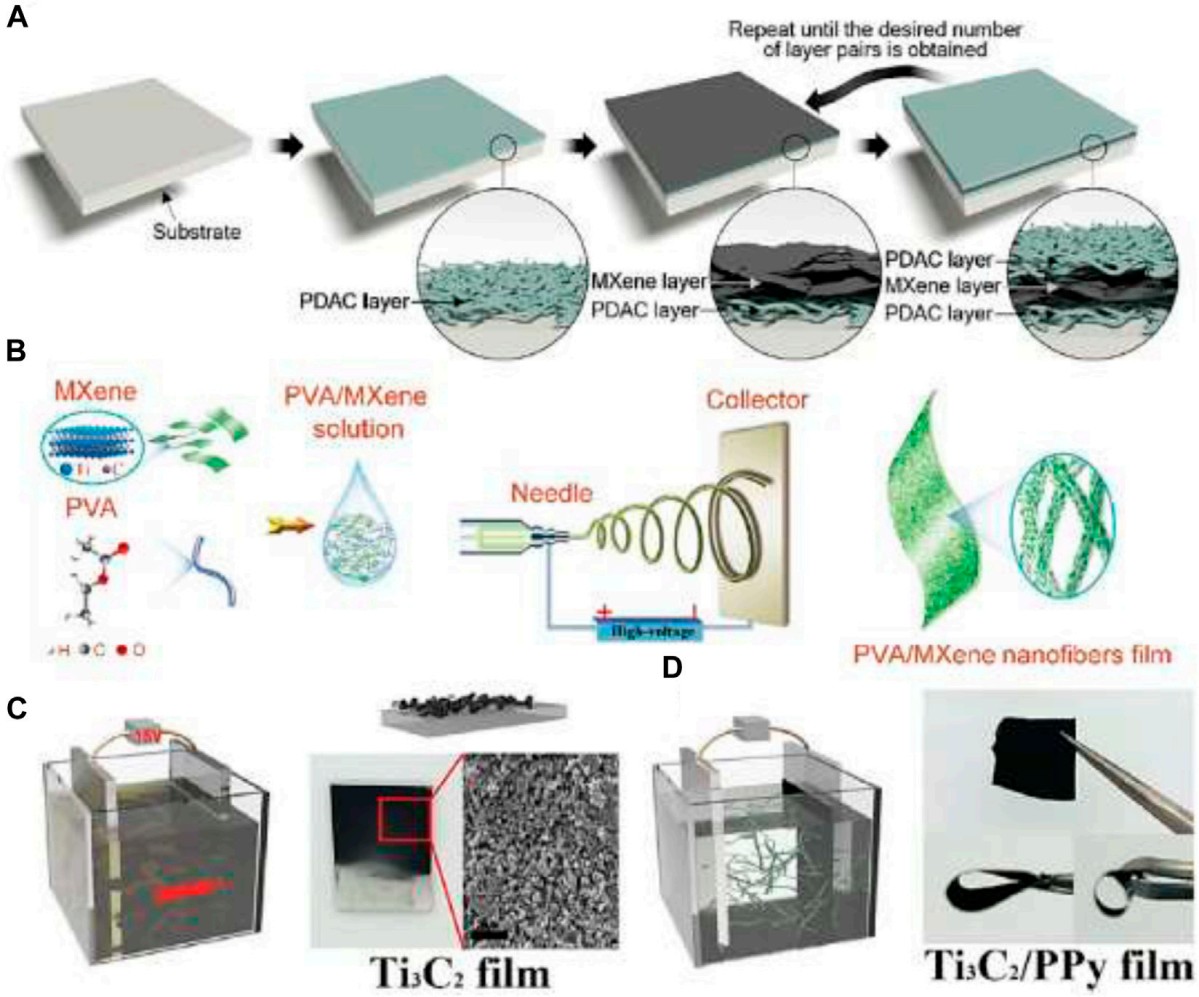
**(A)** Schematic illustration of the synthesis of MXene/PDAC membrane by LbL assembly process. **(B)** Schematic illustration of the fabrication process of MXene/PVA film by electrospinning approach. **(C)** Schematic illustration of the electrophoretic deposition of Ti_3_C_2_ flakes. **(D)** Schematic illustration of the synthesis of Ti_3_C_2_/PPy membrane by electrochemical polymerization of PPy on Ti_3_C_2_ film. Panel a: Reprinted from ref (An et al., 2018) ©2016, The Authors, **(B)**: Reprinted with permission from ref (C. Jiang et al., 2019). ©2019 Elsevier. Panels c and d: Reprinted with permission from ref (M. Zhu et al., 2016a). © 2016 John Wiley and Sons. A step-by-step guide for the LbL assembly is as.

This process has precise control over layer arrangement, suitable for a variety of polymers and MXenes. They can be used for thin films and coatings. However, the process is time-consuming and is limited to specific applications due to its layer-to-layer nature. Layer-by-layer (LbL) assembly (Figure 8) is a versatile technique for fabricating MXene-polymer nanocomposites with precise control over layer thickness and composition (Chen et al., 2020).

##### 3.2.5.1 Material and method

The MXene of interest, such as titanium carbide (Ti_3_C_2_T_x_) or others, is prepared through appropriate synthesis methods. The chosen polymer, typically in the form of a solution or dispersion compatible with the assembly process. Polyelectrolytes like poly (allylamine hydrochloride) (PAH) or poly (sodium 4-styrenesulfonate) (PSS) are commonly used. A solvent suitable for both MXene dispersion and polymer dissolution. Common solvents include water, ethanol, or other polar solvents. A solid substrate on which the LbL assembly will take place. Common substrates include silicon wafers, glass slides, or functionalized surfaces. Additives such as crosslinkers or stabilizers may be used to enhance film stability or modify properties.

The MXene is dispersed in the chosen solvent to create a stable MXene dispersion, with sonication or high-shear mixing possibly utilized to achieve uniform dispersion. The chosen polymer is dissolved in the solvent to generate a polymer solution or dispersion, with the polymer concentration adjustable based on desired film properties. The substrate is thoroughly cleaned to remove any contaminants or residues that may affect film formation, and optionally, the substrate surface is functionalized to promote adhesion or enhance film stability. The process is repeated by immersing the substrate in the polymer solution, allowing the polymer to adsorb onto the MXene-coated substrate surface. The substrate is rinsed with solvent to eliminate any unbound polymer molecules and stabilize the polymer layer. Deposition of MXene and polymer layers is continued alternately until the desired number of layers is achieved, with control over the sequence and thickness of layers by adjusting deposition parameters. Post-treatment steps, such as crosslinking or annealing, may be performed to enhance film stability or modify properties. The assembled MXene-polymer nanocomposite film is characterized to evaluate its structure, morphology, and properties, with techniques such as atomic force microscopy (AFM), ellipsometry, or spectroscopic methods employed.

##### 3.2.5.2 Consideration

The pH and ionic strength of the deposition solutions can influence layer deposition and film properties, so these parameters should be controlled and optimized. Parameters such as immersion time, solution concentration, and drying conditions can affect film thickness and quality and should be carefully optimized. The substrate material and surface properties can influence film adhesion, stability, and performance, so substrate selection should be based on the desired application. LbL assembly allows for precise control over film composition, enabling the incorporation of multiple materials and functionalities into the nanocomposite film.

Layer-by-layer assembly offers fine control over film thickness, composition, and functionality, making it a versatile technique for fabricating MXene-polymer nanocomposites with tailored properties for various applications. Optimization of deposition parameters and thorough characterization are essential for achieving desired film properties and performance.

#### 3.2.6 Electrospinning

They produce nanofibrous structures for enhanced properties, are suitable for a variety of polymers, and enable the fabrication of nanocomposite mats (Boys, 1887). However, they require specialized equipment and are limited to certain polymers and MXenes. Integration of electrospinning with other techniques for multifunctional nanocomposites may overcome this difficulty. Electrospinning is a versatile and widely used method for fabricating nanofibrous structures, including MXene-polymer nanocomposites (Ziabicki, 1976).

##### 3.2.6.1 Material and method

After the preparation of the MXene of interest, such as titanium carbide (Ti_3_C_2_T_x_), through an appropriate synthesis method, a polymer is chosen which is typically in the form of a solution or melt suitable for electrospinning. Common polymers include polyvinyl alcohol (PVA), polyethylene oxide (PEO), or others. A solvent suitable for both MXene dispersion and polymer dissolution. Common solvents include water, dimethylformamide (DMF), or other polar solvents. A grounded collector, typically in the form of a rotating drum or flat plate, collects the electrospun fibers. A high voltage power supply capable of generating electric fields in the range of 1–30 kV. A syringe pump or similar device for controlled delivery of the polymer solution during electrospinning. A spinneret or needle with a fine tip, through which the polymer solution is extruded during electrospinning (D. Li and Xia, 2004).

The MXene is dispersed in the chosen solvent to create a stable MXene dispersion, with sonication or high-shear mixing possibly employed to achieve uniform dispersion. The chosen polymer is dissolved in the solvent to form a polymer solution, with the polymer concentration optimized for electrospinning, typically in the range of 5%–20% w/v. The electrospinning apparatus is set up in a controlled environment to prevent airflow disturbances, and the grounded collector is positioned at an appropriate distance (usually 10–20 cm) from the spinneret. The polymer solution is loaded into a syringe or reservoir connected to the spinneret, ensuring it is free of air bubbles to prevent disruption during electrospinning. A high voltage (typically in the range of 1–30 kV) is applied between the spinneret and the collector to generate an electric field, initiating the electrospinning process by dispensing the polymer solution through the spinneret at a controlled flow rate (typically in the range of 0.1–10 mL/h). The electric field induces a charge on the surface of the polymer solution droplet, leading to the formation of a Taylor cone and subsequent elongation into nanofibers. Simultaneously, the MXene dispersion is introduced into the polymer solution or applied directly onto the collector to incorporate MXene into the electrospun fibers. The electrospun fibers are collected onto the grounded collector, forming a nonwoven mat or membrane, with the rotating drum or flat plate possibly used to facilitate continuous fiber collection. Post-treatment steps, such as drying, crosslinking, or annealing, may be performed to enhance fiber stability or modify properties. The electrospun MXene-polymer nanocomposite fibers are characterized to evaluate their structure, morphology, and properties, with techniques such as SEM, transmission electron microscopy (TEM), or mechanical testing employed (Merritt et al., 2012; Keirouz et al., 2020).

##### 3.2.6.2 Consideration

The polymer concentration and solvent composition are optimized to achieve the desired solution viscosity for electrospinning. Control electrospinning parameters such as voltage, flow rate, and distance between the spinneret and collector to obtain uniform and well-aligned fibers. Uniform dispersion of MXene is ensured within the polymer solution to facilitate its incorporation into the electrospun fibers and enhance their properties. Cautions are exercised when working with high voltages and organic solvents, and follow appropriate safety protocols to prevent accidents or injuries.

Electrospinning offers precise control over fiber diameter, morphology, and composition, making it a valuable technique for fabricating MXene-polymer nanocomposite fibers with tailored properties for various applications such as filtration, tissue engineering, and sensors. Optimization of electrospinning parameters and thorough characterization are essential for achieving desired fiber properties and performance.

#### 3.2.7 Chemical vapor deposition

They allow for direct synthesis on substrates and have uniform coatings on various surfaces along with precise control over film thickness. However, they require specialized equipment and are limited to specific substrates. These days, the scalability is improved, and have control over the CVD process. Chemical vapor deposition (CVD) is a method commonly used for the synthesis of thin films or coatings of MXene-polymer nanocomposites on solid substrates (Shareef et al., 1995; Schropp et al., 2000; Hamzan et al., 2021).

##### 3.2.7.1 Material and method

The MXene of interest, such as titanium carbide (Ti_3_C_2_T_x_) or others, is prepared through appropriate synthesis methods. The chosen polymer precursor, typically in the form of a vapor or gas, will react or deposit onto the substrate surface. A solid substrate onto which the MXene-polymer nanocomposite film will be deposited. Common substrates include silicon wafers, glass slides, or functionalized surfaces. A CVD reactor is equipped with the necessary components for gas flow control, temperature control, and vacuum pumping. An inert carrier gas such as nitrogen or argon, is used to transport the polymer precursor vapor to the substrate. Additives such as catalysts or dopants may be used to modify film properties or promote deposition. The substrate is thoroughly cleaned to remove any contaminants or residues that may affect film deposition, with the substrate surface optionally functionalized to promote adhesion or enhance film properties. The MXene powder is loaded onto a substrate holder inside the CVD reactor, ensuring uniform dispersion of MXene particles on the substrate surface. The polymer precursor is prepared in the desired form for CVD deposition, which may involve heating the precursor to its vaporization temperature or introducing it as a gas into the CVD reactor. The CVD reactor is purged with an inert gas (e.g., nitrogen or argon) to remove air and moisture. The polymer precursor vapor or gas is introduced into the CVD reactor at the desired flow rate, and the substrate is heated to the appropriate temperature for polymer deposition, typically in the range of 100°C–300°C. The polymer precursor reacts or deposits onto the substrate surface, forming a thin film or coating. Alternatively, MXene can be simultaneously deposited with the polymer precursor by introducing MXene vapor or gas into the CVD reactor alongside the polymer precursor. Deposition parameters such as temperature, precursor flow rate, and deposition time are controlled to achieve the desired film thickness and properties. Post-treatment steps, such as annealing or surface modification, may be performed to enhance film properties or stability. The deposited MXene-polymer nanocomposite film is characterized to evaluate its structure, morphology, and properties, with techniques such as SEM, X-ray diffraction (XRD), or spectroscopic methods employed.

**FIGURE 9:**
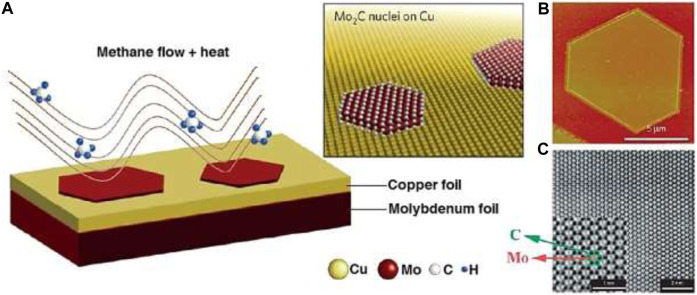
**(A)** The synthesis process of Mo_2_C. **(B)** Images depicting hexagonal ultra-thin Mo_2_C crystals. **(C)** STEM diagrams (P. Hu et al., 2022; Amu-Darko et al., 2024).

A novel method utilizing chemical vapor deposition (CVD) has been uncovered for the direct production of ultrathin MXene materials, offering a new avenue for MXene-based material fabrication (Gogotsi, 2015) (C. Xu et al., 2015; Geng et al., 2017). By employing methane as a carbon source over a Cu/Mo alloyed surface at temperatures surpassing 1085°C (Figures 9A–C) (Gogotsi, 2015) (C. Xu et al., 2015) large-area high-quality 2D ultrathin α-Mo_2_C crystals (−3 nm) were successfully synthesized. Control over crystal size and thickness was achieved through manipulation of experimental conditions, where growth time influenced lateral size while growth temperature affected nucleation density. The method demonstrated versatility in generating various crystal shapes, including triangles, rectangles, hexagons, octagons, nonagons, and dodecagons, all exhibiting hexagonal packing of Mo atoms. Furthermore, enabling the fabrication of ultrathin β-Mo_2_C nanosheets using a rapid and scalable synthesis approach involving MoO_2_ nanosheets as templates and Mo sources. Unlike 2D materials obtained from alternative methods, CVD-fabricated MXenes showcased fewer defects and terminations, facilitating comprehensive investigations into their intrinsic properties and domain boundaries’ effects. The exploration of bottom-up synthetic approaches is encouraged to prepare other types of monolayered MXenes with diverse functionalities, thereby enabling further investigations into their inherent electronic and optical properties (Gogotsi, 2015; Jeon et al., 2018; Sang et al., 2018)(L. Wang et al., 2016).

##### 3.2.7.2 Consideration

A polymer precursor compatible with the CVD process and suitable for deposition onto the substrate surface is chosen and optimized the substrate temperature and deposition parameters to ensure uniform film growth and adhesion. The flow rates of carrier gas and precursor gas are controlled to achieve desired deposition rates and film properties. The deposition conditions are adjusted to control the composition and stoichiometry of the deposited MXene-polymer nanocomposite film.

CVD offers precise control over film thickness, composition, and properties, making it a valuable technique for fabricating MXene-polymer nanocomposites on solid substrates. Optimization of deposition parameters and thorough characterization are essential for achieving desired film properties and performance.

#### 3.2.8 Hydrothermal/solvothermal methods

It is a method of producing a single crystal under high pressure using a hot mineral water solution that is hydrothermal and producing a chemical compound that is solvothermal (Demazeau, 2008). They have a low-temperature synthesis approach that is scalable applicable to various polymers and enables large-scale production. However, the reaction conditions may affect MXene properties and require careful optimization. They are advanced by using environmentally friendly solvents (like water, limonene, ethyl acetate, ethers, esters, ionic liquids, etc.) and improved reaction control (like catalysis, optimization techniques, etc.). Hydrothermal (Byrappa and Yoshimura, 2013) and solvothermal methods are commonly used for synthesizing MXene-polymer nanocomposites in a controlled environment (Rabenau, 1985).

##### 3.2.8.1 Material and method

The MXene of interest, such as titanium carbide (Ti_3_C_2_T_x_) or others, is prepared through appropriate synthesis methods. The chosen polymer precursor, is typically in the form of a solution or dispersion compatible with the hydrothermal/solvothermal conditions. Common polymers include polyvinyl alcohol (PVA), polyethylene glycol (PEG), or others. A solvent is suitable for both MXene dispersion and polymer dissolution under hydrothermal/solvothermal conditions. Common solvents include water, ethanol, or other polar solvents. A sealed autoclave or reaction vessel capable of withstanding high temperatures and pressures. A heating source capable of providing the required temperature for the hydrothermal/solvothermal reaction. Additives such as surfactants, catalysts, or stabilizers may be used to modify reaction kinetics or improve nanocomposite properties.

The MXene is dispersed in the chosen solvent to create a stable MXene dispersion, with sonication or high-shear mixing potentially employed to achieve uniform dispersion. The chosen polymer is dissolved in the solvent to form a polymer solution or dispersion, with the polymer concentration optimized for the hydrothermal/solvothermal conditions. The MXene dispersion and polymer solution are mixed in the desired ratio to obtain a homogeneous MXene-polymer mixture, ensuring thorough mixing to achieve uniform dispersion of MXene within the polymer matrix. The MXene-polymer mixture is transferred into a sealed autoclave or reaction vessel suitable for hydrothermal/solvothermal reactions, ensuring the vessel is clean and free of contaminants. The autoclave or reaction vessel is heated to the desired temperature for the hydrothermal/solvothermal reaction, typically ranging from 100°C to 250°C, depending on the polymer and MXene used. The reaction temperature is maintained for a specified duration to allow for the synthesis of the MXene-polymer nanocomposite. After the reaction is complete, the autoclave or reaction vessel is cooled to room temperature, and the MXene-polymer nanocomposite product is retrieved. The product may be in the form of a gel, powder, or precipitate, depending on the reaction conditions. Post-treatment steps, such as washing, drying, or annealing, may be performed to remove any unreacted precursors or by-products and to enhance the properties of the nanocomposite material. The synthesized MXene-polymer nanocomposite is characterized to evaluate its structure, morphology, and properties, with techniques such as SEM, XRD, or spectroscopic methods employed.

##### 3.2.8.2 Consideration

The reaction temperature, pressure, and duration to achieve the desired properties of the MXene-polymer nanocomposite are optimized. A solvent suitable (like N-methyl-2-pyrrolidone (NMP) a polar aprotic solvent that has good solubility for a wide range of polymers, including many commonly used ones such as polyvinylidene fluoride (PVDF), polyvinyl alcohol (PVA), and polyethylene oxide (PEO)) for both MXene dispersion and polymer dissolution under hydrothermal/solvothermal conditions are chosen. The additives or surfactants are incorporated to control particle size, and morphology, or to enhance the dispersion of MXene within the polymer matrix. High temperatures and pressures are handled with caution and follow appropriate safety protocols during the hydrothermal/solvothermal reaction.

This method offer controlled conditions for synthesizing MXene-polymer nanocomposites with tailored properties. Optimization of reaction parameters and thorough characterization are essential for achieving desired nanocomposite properties and performance.

#### 3.2.9 Emulsion polymerization

Production of hydrophobic polymers in industrial and academic scales (Hohenstein and Mark, 1946) They are suitable for water-soluble polymers and have good control over particle size and distribution. However, they are limited to specific polymer types and may require additional steps for nanocomposite formation. The novel emulsion systems are developed for improved stability. Emulsion polymerization is a versatile method for preparing polymer-MXene nanocomposites in the form of latex particles dispersed in an aqueous medium (Whitby and Katz, 1933).

##### 3.2.9.1 Material and method

The MXene of interest, such as titanium carbide (Ti_3_C_2_T_x_), is prepared through appropriate synthesis methods, and the monomers that are compatible with the emulsion polymerization process are selected for polymerization. Common monomers include styrene, methyl methacrylate, or butyl acrylate. An emulsifier or surfactant stabilizes the emulsion and prevents the coalescence of polymer particles. Common surfactants include sodium dodecyl sulfate (SDS), polyvinyl alcohol (PVA), or cetyltrimethylammonium bromide (CTAB). A water-soluble initiator to initiate the polymerization reaction in the aqueous phase. Common initiators include potassium persulfate (KPS), ammonium persulfate (APS), or hydrogen peroxide. Water is the continuous phase for emulsion polymerization. Optionally, a stabilizer such as polyvinyl alcohol (PVA) or polyethylene glycol (PEG) may be added to improve stability and control particle size.

The MXene is dispersed in water using mechanical agitation or sonication to obtain a stable aqueous dispersion, with the MXene concentration optimized based on the desired loading in the nanocomposite. The emulsion is prepared by mixing the MXene dispersion with the surfactant solution, with the surfactant concentration optimized to ensure the stabilization of the emulsion. The chosen monomer or monomer mixture is dissolved in water-soluble initiators to form the aqueous monomer phase, with the monomer concentration adjustable based on the desired properties of the polymer-MXene nanocomposite. The polymerization reaction is initiated by adding the monomer solution to the emulsion under stirring or agitation, with the addition performed gradually to control the particle size and nucleation rate. The reaction temperature is maintained, and stirring continues until the polymerization is complete, typically controlled below the boiling point of water. During polymerization, MXene particles become embedded within the growing polymer particles, forming a polymer-MXene nanocomposite. The surfactant molecules at the interface between water and monomer droplets stabilize the growing polymer particles, preventing coalescence. After polymerization, the nanocomposite latex can undergo post-polymerization treatments such as purification, drying, or chemical modification to remove unreacted monomers or improve their properties. The synthesized polymer-MXene nanocomposite is characterized to evaluate its structure, morphology, and properties, with techniques such as dynamic light scattering (DLS), TEM, or mechanical testing employed.

##### 3.2.9.2 Consideration

An appropriate surfactant is selected to stabilize the emulsion and control particle size during polymerization. Select monomers compatible with water-based emulsion polymerization and capable of forming stable latex particles. Water-soluble initiators are used for polymerization in the aqueous phase, ensuring efficient initiation and control of the reaction. Maintain appropriate stirring and temperature conditions throughout the polymerization process to ensure uniform particle formation and polymerization kinetics.

Emulsion polymerization offers advantages such as easy scalability, control over particle size, and compatibility with aqueous systems, making it a promising method for preparing polymer-MXene nanocomposites in latex form. Optimization of parameters and thorough characterization are essential for tailoring the properties of the nanocomposite for specific applications.

Each synthesis method has its own set of considerations, and the choice depends on the specific requirements of the application. Recent advancements often focus on improving scalability, efficiency, and control over the nanocomposite’s structure and properties. Exploration is going on innovative approaches to enhance the performance and broaden the applicability of MXene-polymer nanocomposites in diverse fields.

#### 3.2.10 Characterization

Techniques such as XRD and TEM are used to confirm the 2D structure and layer spacing of MXenes. X-ray photoelectron spectroscopy (XPS) and FTIR help analyze the chemical composition and functional groups on the MXene surface. Brunauer-Emmett-Teller (BET) surface area analysis is commonly used to determine the specific surface area of MXenes, providing insights into their porosity.

MXene synthesis methods are continuously evolving, with an exploration of new etchants, delamination techniques, and polymer integration strategies to enhance the properties and applications of these unique materials. The ability to customize MXene structures and incorporate them into nanocomposites opens up a wide range of possibilities for various technological advancements.

## 4 Biomedical application

There are several fields under the biomedical application of MXene Polymer nanocomposite (George, Tandon, and Kandasubramanian, 2020; Garg and Ahmad, 2022; Parajuli et al., 2022).

### 4.1 Role in drug delivery systems

**FIGURE 10:**
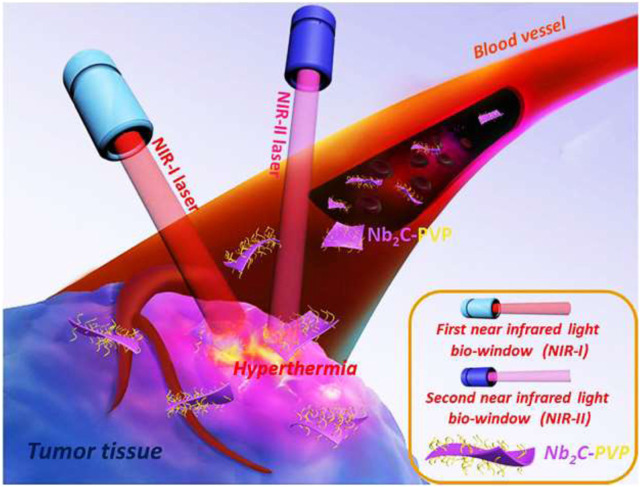
A sketch of Nb_2_C/PVP used for tumor ablation by *In Vivo* Photothermal irradiated with NIR-I and NIR-II (Reprinted with permission from ref (Lin, Gao, et al., 2017). Copyright 2017 American Chemical Society.

MXene-polymer nanocomposites play a significant role in drug delivery systems due to their unique properties of higher photothermal conversion and pH sensitivity, which offer advantages for enhancing therapeutic efficacy and addressing challenges associated with conventional drug delivery methods. A sketch of Nb_2_C/PVP used for tumor ablation by *In Vivo* Photothermal irradiated with NIR-I and NIR-II is shown in Figure 10. Here are some key roles of MXene-polymer nanocomposites in drug delivery systems.

#### 4.1.1 Improved drug loading and release

**FIGURE 11:**
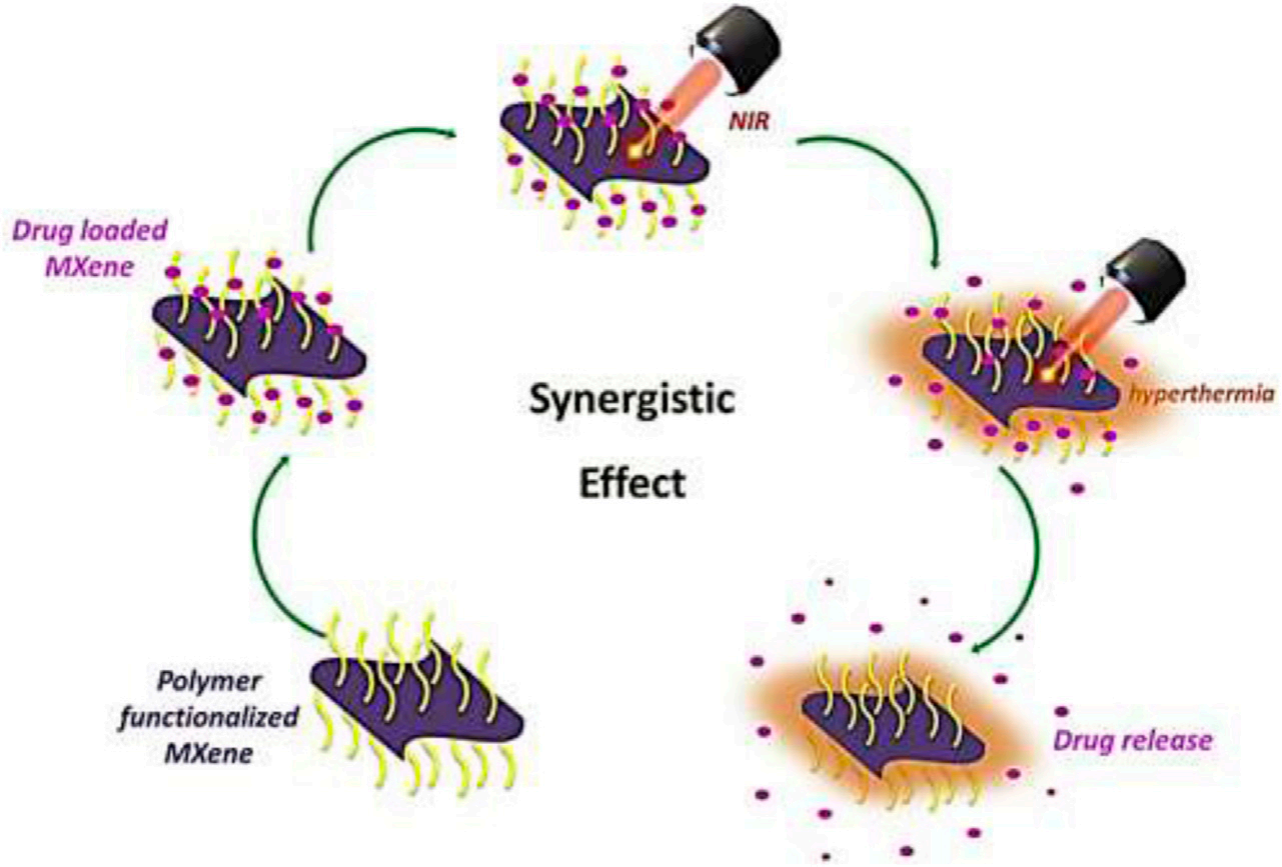
The chemo and photothermal therapy process showing synergistic effect.

MXene nanosheets provide a high surface area and abundant functional groups, allowing for efficient loading of therapeutic agents such as drugs, proteins, or nucleic acids (Sur et al., 2019). The controlled release of these agents can be achieved by modulating the interaction between the drug molecules and the nanocomposite matrix, leading to sustained or triggered release profiles (P. Zhang et al., 2019). For example: the synergistic effect developed in SP surface modified with Ti_3_C_2_ destroys the cancerous cells with the help of NIR laser (X. Han et al., 2018) as shown in Figure 11.

#### 4.1.2 Enhanced biocompatibility and stability

Incorporating MXenes into polymer matrices can improve the biocompatibility and stability of drug delivery systems. The polymer coating protects the MXene nanosheets from degradation and minimizes potential cytotoxicity, enhancing the overall biocompatibility of the nanocomposite (Zhao et al., 2021; Cui et al., 2023).

#### 4.1.3 Targeted drug delivery

Surface functionalization of MXene-polymer nanocomposites allows for the introduction of targeting ligands such as antibodies, peptides, or aptamers. These ligands can selectively bind to specific receptors or biomarkers overexpressed on target cells, facilitating targeted drug delivery and minimizing off-target effects (Mohajer et al., 2022) (A. Liu et al., 2022). G. Liu et al. and Z. Li et al. studied MXene nanocomposites for the targeted drug delivery and found efficient for that. (Z. Li et al., 2018).

#### 4.1.4 Responsive drug release

MXene-polymer nanocomposites can be engineered to respond to external stimuli such as pH, redox, temperature, light, electric, magnetic, etc. By incorporating responsive polymers or responsive MXene coatings, drug release from the nanocomposite can be triggered or modulated in a controlled manner, enabling on-demand drug delivery and localized therapy (He et al., 2024).

**FIGURE 12:**
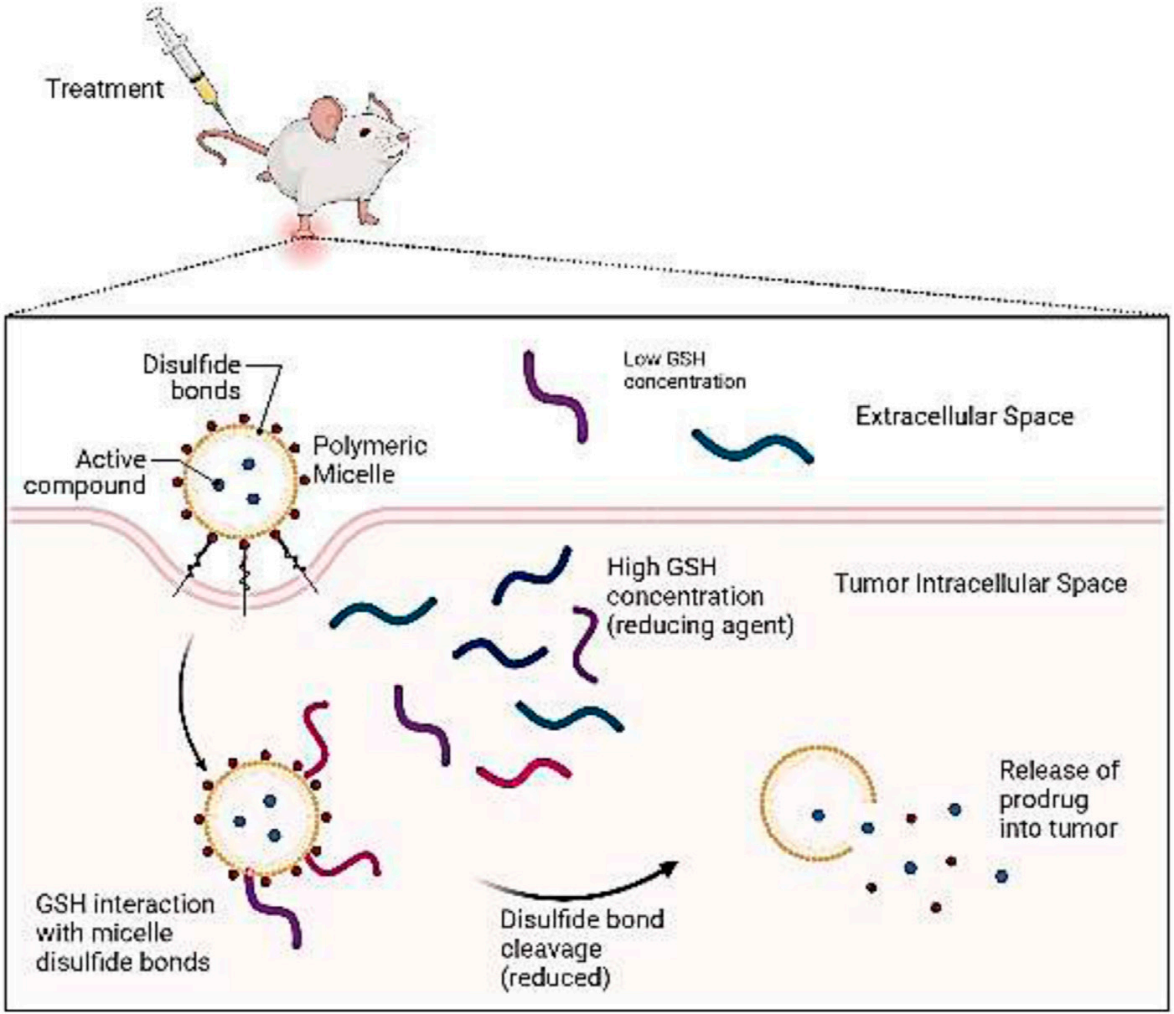
Intra-tumoral Redox responsive drug delivery systems activated by GSH.

The pH of cancerous tissues is lower than normal (i.e. below 7.4) dealing with hydrophilic and hydrophilic interaction. (Lopes et al., 2013). In redox response, there is oxidation-reduction in extra and intracellular regions. For example: glutathione (GSH) can be the reducing agent as shown in Figure 12. (Hatakeyama, 2017). (Y. Dai, Chen, and Zhang, 2018). Some of the responsive polymers are Poly acrylic acid (PAA), polyethylene glycol (PEG), poly 2-dimethylamino ethyl methacrylate (PDMAEMA), polyethylene oxide (PEO), poly oligo ethylene methacrylate (POEGMA), etc. In electro-responsive drug release, the electrically conductive MXene by using the external electric field (Zavahir et al., 2020). Similarly, the external magnetic field plays the same role in magnetic-responsive drug release (Darroudi et al., 2023).The light-responsive molecules like photochromic compounds stimulated by light of a certain wavelength create structural changes in MXene or its surroundings for light-responsive drug release (Zavahir et al., 2020). In case of temperature-responsive drug release, MXene nanosheets into a polymer matrix with a lower critical solution temperature (LCST), the drug release can be triggered by changes in temperature above or below the LCST can be utilized (Karimi et al., 2016).

#### 4.1.5 Theranostic application

MXene-polymer nanocomposites have shown promise for theranostic applications, combining therapeutic and diagnostic functionalities within a single platform. The efficient breast cancer theranostic was achieved by tantalum carbide MXene composite (Z. Liu et al., 2018; Lin et al., 2018). Polyoxometalate as functionalizing agent for Tantalum carbide was studied by L Zong et al., 2018 for theranostic application. MXene Functionalization with imaging agents or contrast agents enables real-time monitoring of drug distribution and therapeutic response, facilitating personalized medicine and treatment optimization (Sivasankarapillai et al., 2020; Aslam et al., 2023; Nikazar, Mofidi, and Mortazavi, 2023).

#### 4.1.6 Synergistic therapeutic effect

The combination of MXenes with polymers can lead to synergistic therapeutic effects, where the unique properties of each component complement each other. For example, A. Liu et al., 2022, Yang et al., 2022, W Tang et al., 2019 found that MXene’s high conductivity and mechanical strength combined with the polymer’s biocompatibility and versatility can enhance the overall performance of drug delivery systems, enabling novel therapeutic strategies (A. Liu et al., 2022; Yang et al., 2022)(W. Tang et al., 2019).

#### 4.1.7 Multifunctional platforms

MXene-polymer nanocomposites can serve as multifunctional platforms for combination therapy, allowing for the co-delivery of multiple drugs or therapeutic agents with different mechanisms of action. This approach can overcome drug resistance, improve treatment outcomes, and reduce side effects compared to single-agent therapy. Several studies and experiments have demonstrated the efficacy of MXene-polymer nanocomposites in Drug delivery systems. Wang et al. (2019) developed a pH-responsive drug delivery system based on MXene-polymer nanocomposites for targeted cancer therapy. The nanocomposites exhibited high drug loading capacity, controlled drug release behavior, and enhanced cytotoxicity against cancer cells compared to free drugs (Wang et al., 2019). In another study, Liu et al. (2021) synthesized MXene-polymer nanocomposites for the co-delivery of doxorubicin (DOX) and indocyanine green (ICG) for combined chemotherapy and photothermal therapy. The nanocomposites showed improved therapeutic efficacy and enhanced tumor inhibition in a mouse xenograft model compared to monotherapy (X. Liu et al., 2021).

#### 4.1.8 Anticancer treatment

In cancer treatment, therapeutics such as PLGA/Ti_3_C_2_ (Lin, Wang, et al., 2017) are employed for photothermal ablation. Additionally, Ti_3_C_2_/Al has been utilized for cancer treatment under 808 nm laser radiation (Z. Liu et al., 2018). V_2_C nanosheets demonstrate efficacy as photothermal agents for photothermal treatment, with applications in photoacoustic (PA) and magnetic resonance imaging (MRI) (Zada et al., 2020). Moreover, AIPH@Nb_2_C@mSiO_2_ nanocomposites have been employed for thermodynamic therapy targeting cancer cells deficient in oxygen (Xiang et al., 2019). Ti_3_C_2_-DOX complexes have been demonstrated to generate reactive oxygen species (ROS) during photodynamic therapy, effectively targeting and killing cancerous cells (K. Huang et al., 2018). Additionally, they find utility in drug delivery applications. Nb_2_C/polymer nanocomposites have been utilized to ablate tumors via photothermal processes, particularly in the near-infrared region (Lin, Gao, et al., 2017). Furthermore, MnO_x_/Ti_3_C_2_-SP and MnO_x_/Ta_4_C_3_-SP MXene nanocomposites have been employed for the treatment of acidic tumors (C. Dai, Chen, et al., 2017; Lin, Chen, and Shi, 2018).

Thus, MXene-polymer nanocomposites hold great promise for revolutionizing drug delivery systems by offering tailored properties, enhanced functionality, and improved therapeutic outcomes. Continued research and development in this field are expected to lead to the translation of these nanocomposites into clinically relevant applications for the treatment of various diseases.

### 4.2 Imaging and diagnostics

MXene-polymer nanocomposites hold significant potential in imaging and diagnostics due to their unique properties and versatile functionalities. For example: They are used for the allocation of affected areas like tumor regions and monitoring the treatment effect by MRI. Here are some key aspects highlighting their potential in this field.

#### 4.2.1 Enhanced contrast agents

MXene nanosheets can be functionalized with imaging agents such as fluorophores, magnetic nanoparticles, or radioisotopes to enhance contrast in various imaging modalities including optical imaging, MRI, and nuclear imaging techniques (e.g., positron emission tomography (PET) or single-photon emission computed tomography (SPECT)). The integration of MXenes into polymer matrices ensures stability and biocompatibility of the contrast agents, enabling their use *in vivo* for non-invasive imaging (Aslam et al., 2023). Ta_4_C_3_-IONP-SP nanocomposites represent a case employed in MRI applications (Z. Liu et al., 2018) as enhanced contrast agent.

#### 4.2.2 Targeted imaging

Surface modification of MXene-polymer nanocomposites with targeting ligands (e.g., antibodies, peptides, or aptamers) allows for specific binding to molecular targets or biomarkers overexpressed in diseased tissues. This targeted approach improves imaging sensitivity and specificity, enabling early detection and precise localization of pathological lesions (Kumar, Barman, and Singh, 2021).

#### 4.2.3 Multimodal imaging

MXene-polymer nanocomposites can be engineered to exhibit multimodal imaging capabilities by combining different imaging agents within a single platform. For example, integrating fluorescent dyes for optical imaging with magnetic nanoparticles for MRI or radionuclides for PET allows for complementary information acquisition and improved diagnostic accuracy (Z. Liu et al., 2018).

#### 4.2.4 Responsive imaging

MXene-based nanocomposites can respond to external stimuli such as pH, temperature, or specific biomolecules, leading to changes in their imaging properties. C. Dai et al., 2017 used the nanocomposite in pH-guided MRI for hyperthermia treatment of tumors (C. Dai, Lin, et al., 2017). Responsive imaging agents enable dynamic monitoring of physiological processes or disease progression in real-time, providing valuable insights into treatment response and disease mechanisms (X. Han et al., 2018).

#### 4.2.5 Theranostic application

MXene-polymer nanocomposites have the potential for theranostic applications, combining diagnostic and therapeutic functionalities within a single platform. By incorporating both imaging agents and therapeutic agents, these nanocomposites enable image-guided therapy, personalized treatment planning, and monitoring of therapeutic response in real-time (Lin et al., 2018).

#### 4.2.6 Bioimaging

MXene-polymer nanocomposites exhibit excellent biocompatibility and low cytotoxicity, making them suitable for biomedical imaging applications. These nanocomposites can be used for *in vitro* cell imaging, *in vivo* molecular imaging, and bioimaging of tissues and organs, providing valuable information for disease diagnosis, prognosis, and treatment evaluation (Gomez et al., 2018).

#### 4.2.7 Flexible and functional platforms

MXene-polymer nanocomposites offer flexibility in design and functionalization, allowing for the customization of imaging properties based on specific application requirements. Tailoring the composition, size, and surface chemistry of the nanocomposites enables fine-tuning of imaging performance and optimization for different imaging modalities (Ling et al., 2014).

Some additional prominent research was made in Imaging and diagnostics. Xu et al. (2020) developed MXene-polymer nanocomposites as dual-modal contrast agents for both photoacoustic imaging (PAI) and MRI. The nanocomposites exhibited excellent imaging performance with high signal intensity and superior contrast enhancement in tumor imaging (Xu et al., 2020). Kong and Chen in 2022 reported the synthesis of MXene-polymer nanocomposites as theranostic agents for combined photothermal therapy (PTT) and photodynamic therapy (PDT) of cancer (Kong and Chen, 2022). The nanocomposites showed efficient tumor ablation and simultaneous imaging capability for guiding therapy (H. Li et al., 2021).

### 4.3 Biosensors

Biosensors are employed to identify specific elements within the human body. These devices consist of several components: a sensing element, a transducer, and a data interpreter (Prajapati and Kandasubramanian, 2019). The sensing element, typically an immobilized biomolecule like an enzyme, can detect the concentration of a target analyte in its vicinity, generating a biochemical signal. This signal is then converted into an electrical signal by a transducer and subsequently interpreted by a data interpreter. In a study by Rakhi and colleagues, they developed an enzymatic biosensor (GOx/Au/Ti_3_C_2_/Nafion/GCE) for detecting glucose (Rakhi et al., 2016). Glucose is converted into gluconolactone and hydrogen peroxide (H_2_O_2_) through the action of the enzyme glucose oxidase (GOx). The presence of glucose leads to the production of peroxide, generating a high potential indicative of glucose levels. Nafion facilitates the enzyme’s adhesion to the glassy carbon electrode (GCE) (Harper and Anderson, 2010).

In Biosensing and detection, Li et al. (2020) synthesized MXene-polymer nanocomposites for glucose biosensing applications. The nanocomposites exhibited high sensitivity, excellent selectivity, and rapid response toward glucose detection, showing promise for diabetes management and point-of-care testing (X. Li, Zhao, and Wu, 2020). Jiang et al. (2021) developed MXene-polymer nanocomposites for ultrasensitive detection of circulating tumor DNA (ctDNA) in blood samples. The nanocomposites demonstrated high specificity and sensitivity for ctDNA detection, offering potential for early cancer diagnosis and prognosis monitoring (Y. Jiang et al., 2021). The GOx/Au/Ti3C2/Nafion/GCE configuration represents an enzymatic biosensor for glucose detection (Rakhi et al., 2016).

### 4.4 Tissue engineering

In Tissue engineering and regenerative medicine, Zhu et al. (2020) fabricated MXene-polymer nanocomposite scaffolds for bone tissue engineering applications. The nanocomposite scaffolds exhibited excellent biocompatibility, enhanced mechanical properties, and promoted osteogenic differentiation of mesenchymal stem cells (MSCs) *in vitro* (J. Zhu et al., 2020). In a study by Ma et al. (2021), MXene-polymer hydrogels were developed as injectable scaffolds for cartilage repair. The nanocomposite hydrogels exhibited excellent biocompatibility, controlled drug release, and promoted chondrogenic differentiation of MSCs, showing potential for cartilage regeneration (Ma et al., 2021)

The literature demonstrates the biocompatibility of MXene-polymer nanocomposites for biomedical applications, including tissue engineering, drug delivery, and implantable medical devices. Further research is needed to fully understand the biocompatibility mechanisms and optimize the design of MXene-polymer nanocomposites for safe and effective biomedical applications. The synthesis, application, and properties of MXene-polymer nanocomposites are listed in Table 3.

**TABLE 3:** MXene–polymer composite materials for Biocompatibility and environment remedies with synthesis, application, and properties (adopted from (Parajuli et al., 2022)).

S. N	MXene/polymer	Synthesis	Application and properties	Refs
01	AgNP-Ti_3_C_2_T_*x*_/Fe_3_O_4_ and PVA	Electrospinning + heat treatment	Wastewater treatment	Huang et al. (2019)
02	Ti_3_C_2_/PLA	Melt blending	Thermal + mechanical	Xue et al. (2020)
03	Ti_3_C_2_T_*x*_@CS/PU	Dip coating	Pressure sensors	Li et al. (2019)
04	GO_*x*_/Au/Ti_3_C_2_T_*x*_/naflon	Chemical reduction	Biosensors	Rakhi et al. (2016)
05	Ti_3_C_2_T_*x*_/PVDF	Vacuum-assisted filtration	Antibacterial + wastewater treatment	Rasool et al. (2017)
06	Ti_3_C_2_T_*x*_/PDMS	MILD etching	Skin conformal tattoo sensors	Kedambaimoole et al. (2020)
07	Ti_3_C_2_T_*x*_/PEI modified alginate aerogel	Cross-linking reaction	Heavy metal ion absorptions	(Y. Feng et al., 2021)
08	Ti_3_C_2_T_*x*_/PPy	*In situ* depositing	Water Remediation	Shi et al. (2022)
09	Ti_3_C_2_T_*x*_@Au/polydopamine	Polymerization	Photothermal + catalytic activity	Zhang et al. (2022)
10	Ti_3_C_2_T_*x*_/PVDF	MILD etching	Water purification	Jin et al. (2021)
11	Ti_3_C_2_T_*x*_/PA	*In situ* interfacial polymerization	Water desalination	Wang et al. (2020)

Overall, MXene-polymer nanocomposites represent a promising platform for advancing imaging and diagnostics, offering enhanced contrast, targeted imaging, multimodal capabilities, responsiveness, and biocompatibility. Continued research and development in this area are expected to lead to the translation of these nanocomposites into clinically relevant imaging agents for early disease detection, precise diagnosis, and personalized medicine.

These studies highlight the diverse applications and promising outcomes of MXene-polymer nanocomposites in biomedical research, ranging from drug delivery systems and imaging diagnostics to tissue engineering and biosensing. Continued exploration and innovation in this field are expected to lead to the development of novel nanocomposite-based technologies for improved healthcare and disease management.

## 5 Biocompatibility and toxicity

MXene-polymer nanocomposites hold great promise for biomedical applications due to their unique properties and versatile functionalities. Biocompatibility refers to the ability of a material to perform its desired function without eliciting an adverse reaction in living organisms. In the context of MXene-polymer nanocomposites, assessing biocompatibility involves evaluating how these materials interact with biological systems, including cells, tissues, and organs. Several factors influence nanocomposites’ biocompatibility, including the materials’ physicochemical properties, surface characteristics, and degradation behavior (Parajuli et al., 2022). Toxicity assessment is another critical aspect, focusing on the potential adverse effects of MXene-polymer nanocomposites on biological systems. Toxicity can arise from various sources, such as the release of toxic ions, generation of reactive oxygen species (ROS), or physical damage to cells and tissues. Understanding the toxicity profile of nanocomposites is essential for ensuring their safety in biomedical applications, such as drug delivery, tissue engineering, and medical implants (Vasyukova et al., 2022).

Various techniques are being employed to evaluate the biocompatibility and toxicity of MXene-polymer nanocomposites, including *in vitro* assays using cell cultures, *in vivo* studies using animal models, and computational modeling approaches. These studies assess parameters such as cell viability, proliferation, inflammation, oxidative stress, genotoxicity, and organ function following exposure to nanocomposite materials (Khatri et al., 2013). Additionally, surface modification strategies can be employed to enhance the biocompatibility of MXene-polymer nanocomposites by reducing potential cytotoxicity and improving their interactions with biological systems. Surface functionalization with biocompatible molecules or polymers can mitigate adverse effects and promote desirable biological responses.

Ensuring the biocompatibility of these nanocomposites is crucial for their safe and effective use in biological systems. Ti_3_C_2_-SP, Ta_4_C_3_-SP, and MnOx/Ti_3_C_2_-SP are utilized in a photoacoustic signal approach, leveraging stress waves from tissues irradiated by NIR. Ta_4_C_3_-IONP-SPs and Ta_4_C_3_-SP nanocomposites possess the capability to attenuate X-rays, making them suitable for employment in computed tomography (CT) (Z. Liu et al., 2018; C; Dai, Chen, et al., 2017; Lin et al., 2018).

Several studies have investigated the biocompatibility of MXene-polymer nanocomposites, demonstrating their potential for various biomedical applications.

### 5.1 Biocompatibility assessment

Research by Li et al. (2021) conducted comprehensive biocompatibility assessments of MXene-polymer nanocomposites for biomedical applications. The study evaluated cytotoxicity, hemocompatibility, and *in vivo* biocompatibility, demonstrating minimal adverse effects and high biocompatibility of MXene-polymer nanocomposites (X. Li et al., 2021).

### 5.2 Cell viability and proliferation

Wang et al. (2020) investigated the effect of MXene-polymer nanocomposites on cell viability and proliferation for tissue engineering applications. The study showed that MXene-polymer nanocomposites supported cell attachment, proliferation, and differentiation, indicating their biocompatibility and potential for promoting tissue regeneration (Wang et al., 2020). The colloidal solution of Ti_3_C_2_ facilitates the proliferation of Gram-negative *E. coli* and Gram-positive *Bacillus subtilis*, demonstrating antibacterial properties (Han et al., 2017). Ag@Ti_3_C_2_@Cu_2_O nanocomposites serve as photocatalysts suitable for antibacterial applications (Feng et al., 2020).

### 5.3 Inflammatory response

Zhang et al. (2021) evaluated the inflammatory response of MXene-polymer nanocomposites *in vitro* and *in vivo*. The study demonstrated low levels of inflammatory cytokine production and minimal tissue inflammation upon exposure to MXene-polymer nanocomposites, highlighting their biocompatibility and suitability for biomedical applications (Zhang, Y., Wang, S., Liu, X., & Chen, 2021).

### 5.4 Tissue compatibility

Xu et al. (2019) investigated the tissue compatibility of MXene-polymer nanocomposites for implantable medical devices. The study showed favorable tissue responses, minimal fibrous encapsulation, and enhanced tissue integration with MXene-polymer nanocomposites, suggesting their potential for long-term implantation *in vivo* (Y. Xu et al., 2019). Ti_3_C_2_T_z_ combined with PLA and octyltriethoxysilane (OTES) exhibits favorable mechanical properties and biocompatibility, aiding in tissue engineering (Zhang et al., 2018; Zhou et al., 2018).

Conclusively, comprehensive evaluation of biocompatibility and toxicity is essential for advancing the development of MXene-polymer nanocomposites for biomedical applications, ensuring their safe and effective use in various healthcare settings.

## 6 Challenges, solutions, and outlooks

**FIGURE 13:**
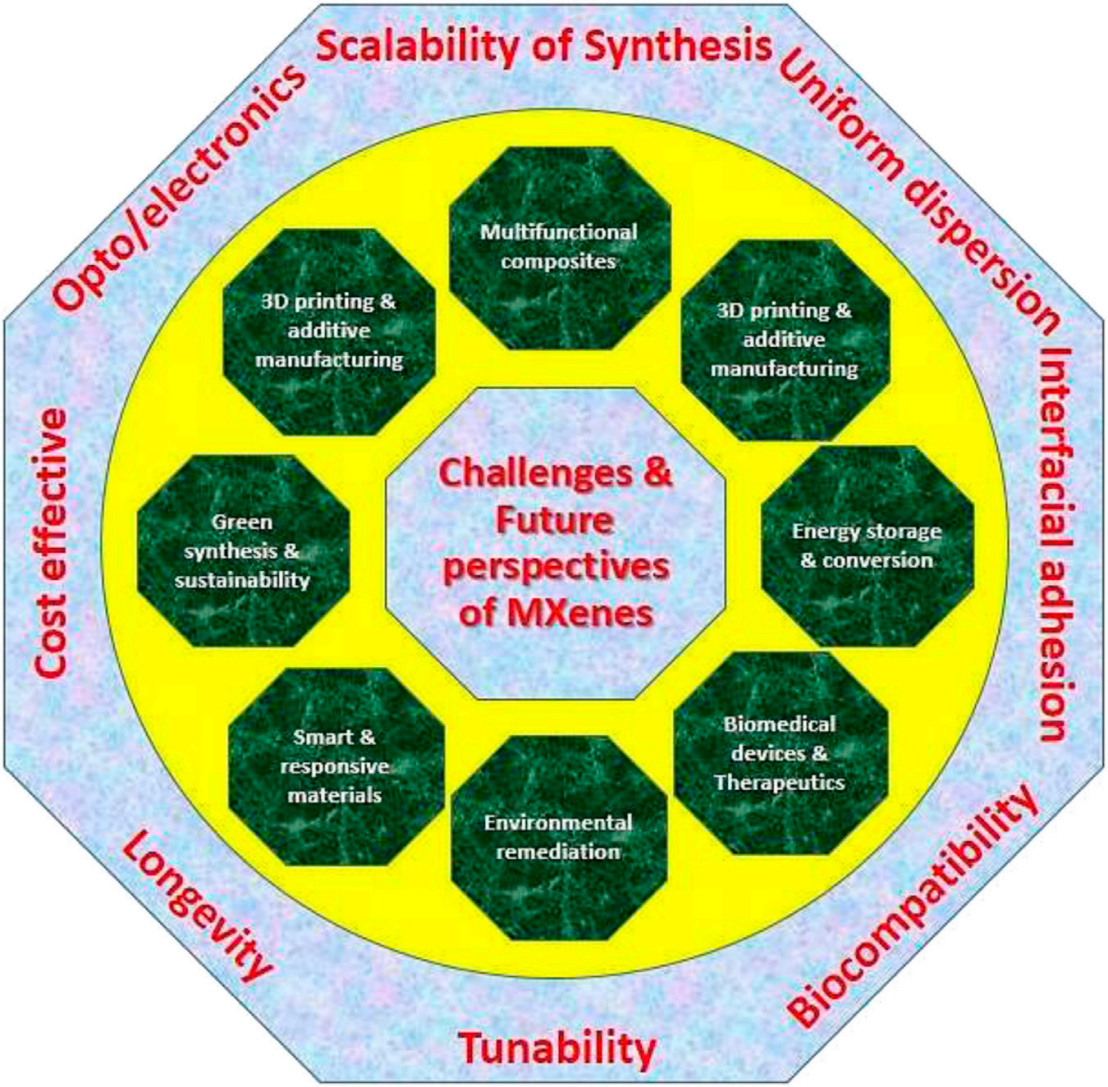
Challenges and Future Perspectives of MXene Polymer nanocomposites.

While MXene-polymer nanocomposites hold immense promise for various applications, several challenges still need to be addressed to fully exploit their potential. The challenges faced by MXenes and their future perspectives are sketched in Figure 13. The challenges are on the outer octagon and the future perspectives in the inner octagon of the Figure. Some of the current challenges in the development and application of MXene-polymer nanocomposites include.

### 6.1 Challenges and solutions

#### 6.1.1 Scalability of synthesis

One major challenge is scaling up the synthesis of MXene-polymer nanocomposites to meet industrial production demands. Current synthesis methods often involve complex and time-consuming procedures, limiting their scalability and commercial viability.

##### 6.1.1.1 Advanced synthesis techniques

Novel synthesis techniques are being employed that enable the scalable production of MXene-polymer nanocomposites with improved efficiency and reproducibility. Techniques such as aerosol-assisted synthesis, microwave-assisted synthesis, and template-assisted synthesis offer potential solutions for overcoming scalability issues.

#### 6.1.2 Uniform dispersion

Achieving uniform dispersion of MXene nanosheets within polymer matrices is crucial for maintaining the desired properties and performance of nanocomposites. However, ensuring uniform dispersion remains a challenge due to the tendency of MXene nanosheets to agglomerate during processing.

##### 6.1.2.1 Surface modification

Surface modification of MXene nanosheets with functional groups or surfactants can enhance their dispersibility within polymer matrices and improve interfacial adhesion. Surface modification techniques such as chemical functionalization, plasma treatment, and surfactant-assisted dispersion are being investigated to address agglomeration and achieve uniform dispersion.

#### 6.1.3 Interfacial adhesion

Achieving strong interfacial adhesion between MXene nanosheets and polymer matrices is essential for enhancing mechanical properties and stability of nanocomposites. However, achieving optimal interfacial adhesion remains challenging due to the inherently different properties of MXenes and polymers.

##### 6.1.3.1 Interfacial engineering

Strategies for optimizing interfacial adhesion between MXene nanosheets and polymer matrices are being explored to enhance the mechanical properties and stability of nanocomposites. Approaches such as compatibilizer incorporation, *in-situ* polymerization, and interfacial coupling agents aim to improve interfacial interactions and enhance nanocomposite performance.

#### 6.1.4 Biocompatibility

While MXene-polymer nanocomposites show great potential for biomedical applications, ensuring their biocompatibility remains a challenge. Further research is needed to comprehensively evaluate the biocompatibility of these nanocomposites and mitigate potential cytotoxicity or immunogenicity issues.

##### 6.1.4.1 Biocompatibility assessment

Ongoing research focuses on comprehensive biocompatibility assessment of MXene-polymer nanocomposites for biomedical applications. Studies involve evaluating cytotoxicity, immunogenicity, and long-term biocompatibility through *in vitro* and *in vivo* experiments, aiming to ensure the safety and efficacy of nanocomposites in biological environments.

#### 6.1.5 Tunable properties

Tailoring the properties of MXene-polymer nanocomposites to meet specific application requirements poses a challenge. Achieving tunable properties such as electrical conductivity, mechanical strength, and thermal stability requires precise control over MXene loading, polymer composition, and processing conditions.

##### 6.1.5.1 Property tuning

Investigations are going on for tuning the properties of MXene-polymer nanocomposites to meet specific application requirements. Strategies such as controlling MXene loading, varying polymer composition, and adjusting processing conditions enable the customization of nanocomposite properties such as electrical conductivity, mechanical strength, and thermal stability.

#### 6.1.6 Longevity

Ensuring the long-term stability of MXene-polymer nanocomposites under various environmental conditions is essential for their practical applications. However, issues such as degradation, leaching, and agglomeration may affect the stability and performance of nanocomposites over time.

##### 6.1.6.1 Stability enhancement

Ongoing research focuses on enhancing the long-term stability of MXene-polymer nanocomposites under various environmental conditions. Strategies such as surface passivation, encapsulation, and cross-linking aim to improve nanocomposite stability, preventing degradation, leaching, and agglomeration over time.

#### 6.1.7 Cost-effectiveness

The cost-effectiveness of MXene-polymer nanocomposites remains a concern, especially for large-scale applications. The high cost of MXene precursors and complex synthesis processes may hinder their widespread adoption in commercial applications. Efforts are underway to develop cost-effective synthesis routes and utilize more affordable precursor materials to reduce the production cost of MXene-polymer nanocomposites. Additionally, process optimization and scale-up strategies aim to streamline production processes and lower overall manufacturing costs.

**FIGURE 14:**
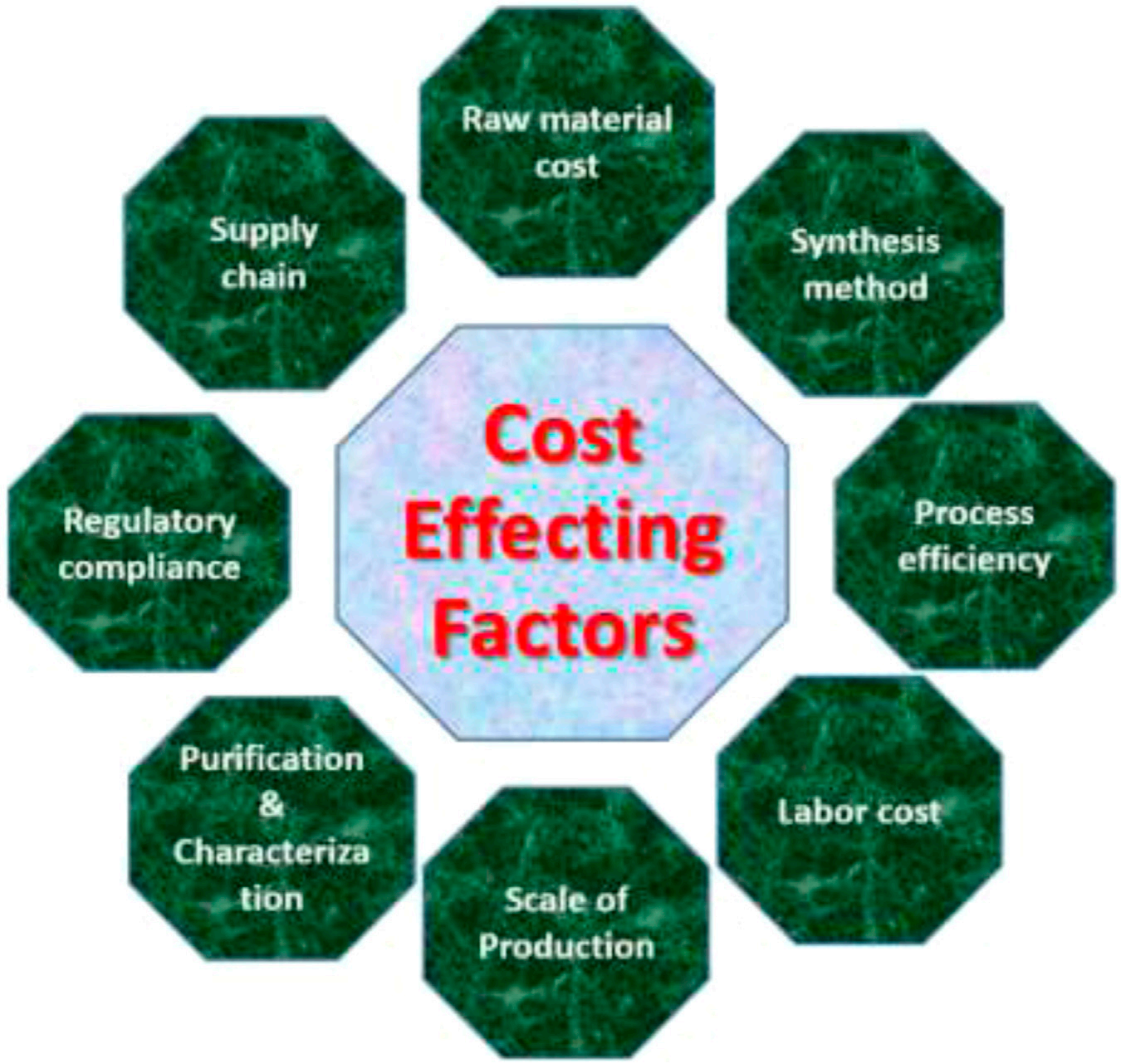
Cost effecting factors of MXene Polymer Nanocomposites.

Since the cost is a major factor in the manufacture of the MXene-polymer nanocomposite on a large scale (Zaed et al., 2024), we have further gone in with the factors of impact as sketched in Figure 14. These factors are.

##### 6.1.7.1 Raw material costs

The cost of raw materials, particularly the precursors needed to produce MXenes, significantly impacts the overall synthesis cost. Precursors may include transition metal carbides, nitrides, or carbonitrides, which can be expensive depending on their source and availability (Rasid et al., 2017).

• Transition metal carbides, nitrides, or carbonitrides: Prices can vary based on factors such as purity, synthesis method, and availability. For example, the cost of titanium carbide powder can range from $50 to $200 per kilogram depending on quality and quantity purchased.
• Precursor chemicals: Prices vary widely depending on the specific chemicals used in the synthesis process. For instance, hydrochloric acid (HCl), a common etchant used in MXene synthesis, costs approximately $0.30 to $1.00 per liter.

##### 6.1.7.2 Synthesis methodology

The choice of synthesis method plays a crucial role in determining costs (Shuck et al., 2021). Some synthesis routes may require expensive equipment, specialized facilities, or energy-intensive processes, increasing production expenses. Optimizing synthesis methods to minimize energy consumption or utilizing simpler techniques can help reduce costs (Jolly, Paranthaman, and Naguib, 2021). High-temperature synthesis methods such as MAX phase etching and exfoliation may require energy-intensive processes, increasing operational costs. For example, the energy cost for heating furnaces or reactors can be significant, ranging from several hundred to thousands of dollars per batch depending on the scale and duration of the synthesis process. Alternatively, low-temperature synthesis methods, such as the use of ionic liquids or mild etchants, may reduce energy consumption but could require specialized equipment or reagents, impacting upfront capital costs (Jolly, Paranthaman, and Naguib, 2021).

##### 6.1.7.3 Process efficiency

The efficiency of the synthesis process affects the yield of desired MXene-polymer nanocomposites per unit of input materials. Low efficiency may result in the wastage of raw materials, energy, and time, driving up production costs. Improving process efficiency through better reaction conditions, catalysts, or purification methods can lower overall costs (Tan et al., 2024).

• Yield of desired MXene product: Efficiency in converting precursor materials into high-quality MXene affects production costs. Higher yields reduce material wastage and associated costs.
• Time required for synthesis: Longer reaction times or complex purification processes can increase labor and energy costs. Optimizing reaction conditions and purification steps can improve process efficiency and reduce overall production time and costs.

##### 6.1.7.4 Labor costs

Skilled labor is essential for synthesizing MXene-polymer nanocomposites, especially in research and development phases where experimentation and optimization are common. Labor costs can significantly impact the overall synthesis cost, particularly if extensive manual intervention is required. Automation and streamlining of processes can help mitigate labor expenses (Islam and Shazali, 2011).

• Skilled labor wages: Labor costs vary widely depending on location, expertise required, and labor market conditions. For example, researchers or technicians involved in MXene synthesis may earn salaries ranging from $40,000 to $100,000 or more annually, depending on experience and qualifications.
• Automation and process optimization: Investments in automation technologies and streamlined processes can reduce labor requirements and associated costs over time.

##### 6.1.7.5 Scale of production

Economies of scale have a significant impact on synthesis costs, as larger production volumes often lead to reduced unit costs (Shuck et al., 2020a). However, expanding production while ensuring consistent quality can be challenging. To achieve cost-effective large-scale production, investments in infrastructure, equipment, and process optimization are essential. The advantages of economies of scale become apparent as production volumes increase, allowing fixed costs to be spread out over a larger output (Shuck et al., 2020). This typically results in lower unit costs. Nevertheless, the initial investments required to scale up production facilities and equipment can be considerable. For instance, transitioning from laboratory-scale to pilot-scale or industrial-scale production may necessitate investments ranging from thousands to millions of dollars. The magnitude of investment depends on factors such as the complexity of the process and the desired output capacity (Charitidis et al., 2014).

##### 6.1.7.6 Purification and characterization

Purification steps to remove impurities and characterization techniques to assess product quality add to the synthesis cost. These processes require additional materials, equipment, and labor. Developing efficient purification methods and adopting cost-effective characterization techniques can help manage these expenses (H. Tang et al., 2021).

• Cost of purification agents and equipment: Purification processes, such as acid washing or filtration, require chemicals, solvents, and specialized equipment. For example, the cost of membrane filtration systems for removing impurities can range from a few hundred to several thousand dollars depending on capacity and efficiency.
• Analytical techniques: Characterization methods such as SEM, XRD, and atomic force microscopy (AFM) incur costs for instrument purchase, maintenance, and consumables. For example, an SEM instrument may cost anywhere from $100,000 to over $1 million, plus ongoing maintenance and operation expenses.

##### 6.1.7.7 Regulatory compliance

This is to follow with environmental, health, and safety regulations adds to the cost of synthesis (Kumari et al., 2023). Implementing sustainable practices and ensuring compliance with regulations may require investments in waste management, safety measures, and compliance monitoring (Journal and Ondokuzmay 2020). One of the concerns is the environmental and safety regulations. It is compliance with regulations governing chemical handling, waste disposal, and workplace safety may require investments in training, protective equipment, and facility modifications (Foulkes et al., 2020). Costs associated with regulatory compliance can vary widely depending on the jurisdiction and specific requirements (Directorate et al., 2015).

##### 6.1.7.8 Supply chain consideration

The availability and cost of ancillary materials, such as solvents, catalysts, and additives, can impact overall synthesis costs. Fluctuations in prices or supply chain disruptions can affect production economics. Diversifying supply sources or developing alternative materials can mitigate these risks (Lele, 2023).

• Ancillary materials and consumables: Prices of solvents, catalysts, and other consumables used in the synthesis process can fluctuate based on market conditions and availability. Bulk purchasing or negotiating long-term supply contracts may help stabilize costs.
• Supply chain disruptions: Events such as natural disasters, geopolitical tensions, or trade restrictions can disrupt the availability of raw materials or increase transportation costs. Diversifying supply sources and maintaining contingency plans can mitigate these risks.

Addressing these critical factors through research, innovation, and process optimization is essential for achieving cost-effective synthesis of MXene-polymer nanocomposites on a large scale. Collaboration between academia, industry, and government stakeholders can accelerate progress in this field and facilitate the commercialization of MXene-based materials.

#### 6.1.8 Regulatory approval

Not only is the cost of manufacturing the nanocomposite, but it is also concerned with obtaining regulatory approval for MXene-polymer nanocomposites, particularly for biomedical and environmental applications, poses a challenge. Comprehensive safety assessments and standardized testing protocols are needed to ensure compliance with regulatory requirements.

##### 6.1.8.1 Regulatory compliance

Investigation are going towards obtaining regulatory approval for MXene-polymer nanocomposites by conducting comprehensive safety assessments and adhering to standardized testing protocols. Collaboration with regulatory agencies and compliance with regulatory guidelines ensure the safe and ethical use of nanocomposites in commercial applications.

Addressing these challenges requires interdisciplinary research focusing on developing novel synthesis techniques, optimizing nanocomposite properties, improving scalability, enhancing biocompatibility, and conducting thorough performance evaluations. Collaborative efforts between academia, industry, and regulatory agencies are essential to overcome these challenges and unlock the full potential of MXene-polymer nanocomposites for diverse applications. Ongoing research in MXene-polymer nanocomposites aims to address the existing challenges through innovative solutions, interdisciplinary collaborations, and advancements in synthesis, characterization, and application techniques. These efforts pave the way for the widespread adoption of MXene-polymer nanocomposites across various industries, including electronics, energy, healthcare, and environmental remediation.

### 6.2 Outlooks

Future directions for the field of MXene-polymer nanocomposites encompass emerging trends and areas for exploration that hold promise for advancing research and applications. Some key future directions include.

#### 6.2.1 Multifunctional nanocomposites

Future research will focus on developing MXene-polymer nanocomposites with multifunctional properties for diverse applications. They are composite materials composed of two or more distinct nanomaterials (such as nanoparticles, nanotubes, or nanosheets) dispersed within a matrix material (polymer, ceramic, or metal). These nanocomposites exhibit a combination of properties not present in the individual components alone. Various synthesis methods are employed to fabricate multifunctional nanocomposites, including solution mixing, *in situ* polymerization, melt blending, electrospinning, and layer-by-layer assembly. Each method offers advantages in terms of control over nanomaterial dispersion, interfacial interactions, and composite structure. These properties can be tailored by adjusting the composition, size, shape, and distribution of the nanomaterials within the matrix. These nanocomposites may integrate functionalities such as conductivity, mechanical strength, electrical conductivity, thermal stability, optical transparency, magnetic response, chemical reactivity, magnetism, biocompatibility, and stimuli-responsiveness, enabling their use in advanced electronics, sensors, actuators, biomedical devices electronics, aerospace, energy storage, sensing, catalysis, and environmental remediation (Yadav, 2023).

Despite their potential, multifunctional nanocomposites face challenges related to scalability, reproducibility, stability, and environmental impact. Future research aims to address these challenges by developing novel synthesis techniques, optimizing composite properties, and exploring new applications in emerging fields.

#### 6.2.2 3D printing and additive manufacturing

The integration of MXene-polymer nanocomposites into additive manufacturing processes, such as 3D printing, will be explored for fabricating complex structures and functional devices with tailored properties. This approach offers opportunities for rapid prototyping, customization, and on-demand manufacturing of MXene-based products.

There are several techniques for 3D printing, including fused deposition modeling (FDM), stereolithography (SLA), selective laser sintering (SLS), binder jetting, and directed energy deposition (DED). Each technique has its advantages and is suitable for different materials and applications (Jiménez et al., 2019). 3D printing has diverse applications across industries such as aerospace, automotive, healthcare, architecture, consumer goods, and education. It is used for prototyping, rapid manufacturing, custom production, tooling, and even creating complex biological structures in biomedical engineering. Some key advantages of 3D printing include rapid prototyping, customization, reduced material waste, design freedom, and the ability to create intricate and lightweight structures. It also allows for on-demand production and decentralized manufacturing.

Despite its numerous advantages, 3D printing still faces challenges related to material limitations, surface quality, printing speed, scalability, and regulatory considerations, particularly in highly regulated industries such as healthcare.

#### 6.2.3 Energy storage and conversion

Future research will focus on leveraging the unique properties of MXene-polymer nanocomposites for energy storage and conversion applications. These nanocomposites may be used as electrode materials in batteries, supercapacitors, and fuel cells, offering high energy density, fast charge-discharge rates, and long-term stability.

MXene-polymer nanocomposites offer several advantages that make them attractive for energy storage and conversion applications (Qiu et al., 2021). These include high surface area, excellent electrical conductivity, mechanical flexibility, and chemical stability. These properties enable them to serve as efficient electrode materials in various energy storage and conversion devices. Furthermore, MXene-polymer nanocomposites have shown promise in enhancing the performance of lithium-ion batteries, fuel cells, and other energy storage devices. Research in this area is ongoing, focusing on optimizing the composition, structure, and processing techniques of MXene-based materials to improve their energy storage and conversion properties (Parajuli et al., 2022).

By leveraging the unique properties of MXene-polymer nanocomposites, future research endeavors seek to develop next-generation energy storage and conversion technologies that offer high energy density, rapid charge-discharge rates, and long-term stability, contributing to the advancement of renewable energy integration and sustainable energy solutions.

#### 6.2.4 Biomedical devices and therapeutics

The development of MXene-polymer nanocomposites for biomedical devices and therapeutics will be explored further. These nanocomposites may find applications in drug delivery systems, tissue engineering scaffolds, biosensors (Guo et al., 2019), and medical implants, offering biocompatibility, bioactivity, and controlled release properties.

The field of biomedical devices and therapeutics is rapidly evolving, driven by technological advancements and innovative research. Here are some future perspectives along.

##### 6.2.4.1 Personalized medicine

Future biomedical devices and therapeutics will increasingly focus on personalized medicine approaches, tailoring treatments to individual patients based on their genetic makeup, lifestyle factors, and disease characteristics. This includes the development of biomarker-based diagnostics, targeted therapies, and precision drug delivery systems (Foroutan, 2015).

##### 6.2.4.2 Implantable medical devices

Advances in materials science, microelectronics, and biocompatible coatings will lead to the development of smarter, more functional implantable devices for monitoring and treating various medical conditions. These devices may incorporate sensors, actuators, and wireless communication capabilities to provide real-time data and therapeutic interventions (Wodlinger, Dweiri, and Durand, 2015).

##### 6.2.4.3 Biomedical devices

Nanomedicine will continue to revolutionize biomedical applications by enabling targeted drug delivery, enhanced imaging, and regenerative therapies. Nanoparticle-based drug carriers, nanofiber scaffolds for tissue engineering, and nanoscale imaging agents hold great potential for improving diagnosis and treatment outcomes (Bai et al., 2006).

##### 6.2.4.4 Bioelectronics and wearable devices

The integration of biology with electronics will lead to the development of advanced wearable devices for continuous health monitoring, disease management, and drug delivery. Flexible and stretchable electronics, bio-compatible materials, and miniaturized sensors will enable seamless integration with the human body (Manjushree et al. 2022).

##### 6.2.4.5 Gene editing and cell therapies

Advances in gene editing technologies such as CRISPR-Cas9 will revolutionize the development of gene therapies for treating genetic disorders and cancer (Jiang and Doudna, 2017). Cell-based therapies, including CAR-T cell immunotherapy and stem cell transplantation, hold promise for regenerative medicine and personalized cancer treatments (H. Wang, La Russa, and Qi, 2016).

In conclusion, the future of biomedical devices and therapeutics holds great promise, with ongoing research and innovation focusing on personalized medicine, implantable devices, nanotechnology, bioelectronics, and gene editing technologies, all aimed at revolutionizing healthcare delivery and improving patient outcomes.

#### 6.2.5 Environmental remediation

Future research will focus on harnessing MXene-polymer nanocomposites for environmental remediation and pollution control. These nanocomposites may be used for water purification, air filtration, soil remediation, and wastewater treatment, offering high adsorption capacity, catalytic activity, and selectivity towards pollutants. One significant future direction involves the integration of emerging technologies such as artificial intelligence, machine learning, and remote sensing into environmental monitoring and remediation strategies. These technologies can improve the efficiency and effectiveness of pollution detection, site characterization, and remediation efforts by providing real-time data, predictive modeling, and automated decision-making capabilities (Shah, Rodriguez-Couto, and Sengor, 2020).

Furthermore, there is growing interest in the development of nature-based solutions and green infrastructure for environmental remediation. These approaches harness natural processes and ecosystems to restore habitats, purify water, and sequester carbon, offering cost-effective and sustainable alternatives to conventional remediation techniques.

#### 6.2.6 Smart and responsive materials

The development of smart and responsive MXene-polymer nanocomposites that can sense and respond to external stimuli, such as light, temperature, pH, and mechanical stress, will be explored. These materials may find applications in smart textiles, adaptive coatings, drug delivery systems, and biomedical implants.

One significant future direction is the enhancement of the stimuli-responsive properties of MXene-polymer nanocomposites through precise control over their composition, structure, and interface interactions. This may involve the incorporation of stimuli-responsive polymers or functionalization of MXene nanosheets with responsive molecules to achieve tunable and reversible responses to environmental changes. Furthermore, research efforts are focused on integrating MXene-polymer nanocomposites into multifunctional devices and systems for diverse applications. These may include smart coatings for corrosion protection, stimuli-responsive drug delivery systems for targeted therapies, and adaptive materials for shape-shifting structures or wearable electronics (Parajuli et al., 2022).

#### 6.2.7 Green synthesis and sustainability

Future research will focus on developing green synthesis routes and sustainable manufacturing processes for MXene-polymer nanocomposites. These efforts aim to reduce environmental impact, minimize resource consumption, and enhance the eco-friendliness of nanocomposite production. The future research in this direction involves the following.

##### 6.2.7.1 Environmentally benign synthesis routes

One focal point of future research involves exploring synthesis pathways that minimize or eliminate hazardous chemicals, volatile organic compounds (VOCs), and other environmentally detrimental substances. Green synthesis methodologies, such as aqueous-based processes or solvent-free techniques, are gaining traction for their reduced environmental footprint and improved safety profiles.

##### 6.2.7.2 Renewable feedstock utilization

Another pivotal aspect revolves around harnessing renewable feedstock and sustainable raw materials for MXene synthesis. By shifting away from fossil fuel-derived precursors towards bio-based or recycled materials, researchers aim to lessen dependence on finite resources and promote circular economy principles.

##### 6.2.7.3 Energy-efficient production process

Efficiency improvements in energy consumption during the synthesis and processing stages represent a crucial avenue for sustainable manufacturing. Integration of energy-saving technologies, waste heat recovery systems, and renewable energy sources can contribute to lowering carbon emissions and enhancing overall energy sustainability.

##### 6.2.7.4 Waste minimization and recycling strategies

Addressing waste generation throughout the production lifecycle is paramount. Implementation of waste minimization techniques, recycling initiatives, and closed-loop manufacturing systems can help reduce environmental burden and optimize resource utilization, thereby fostering a more sustainable nanocomposite manufacturing ecosystem.

##### 6.2.7.5 Life cycle assessment and environmental impact analysis

Comprehensive life cycle assessments and environmental impact analyses play a pivotal role in quantifying the ecological footprint of MXene-polymer nanocomposites. By systematically evaluating the environmental consequences of different production pathways, researchers can identify areas for improvement and guide decision-making towards more sustainable practices (F. Zhang et al., 2022).

##### 6.2.7.6 Regulatory compliance and green certification

Adherence to stringent environmental regulations and attainment of green certifications signify essential benchmarks for sustainable nanocomposite production. Compliance with eco-labeling schemes, such as the EU Ecolabel or Green Seal, can validate adherence to environmental standards and enhance market acceptance of green products.

##### 6.2.7.7 Collaborative research and knowledge sharing

Collaboration among academia, industry, and regulatory bodies is instrumental in advancing green synthesis initiatives and fostering knowledge exchange. Interdisciplinary research consortia, public-private partnerships, and collaborative platforms facilitate innovation, accelerate technology transfer, and drive collective progress towards sustainability goals (Payumo et al., 2020).

By prioritizing green synthesis principles and sustainability-driven manufacturing paradigms, the research community aims to usher in a new era of environmentally responsible MXene-polymer nanocomposite production, thereby harmonizing technological progress with ecological stewardship.

#### 6.2.8 Computational modeling and design

The integration of computational modeling and simulation techniques will play a significant role in the rational design and optimization of MXene-polymer nanocomposites. Computational approaches enable predictive modeling of nanocomposite properties, structure-property relationships, and performance under different conditions.

One significant aspect of computational modeling is its ability to explore the effects of different parameters, such as MXene concentration, polymer morphology, and processing conditions, on the final properties of nanocomposites. This predictive capability allows researchers to optimize material composition and processing parameters to achieve desired performance metrics, such as mechanical strength, electrical conductivity, and thermal stability. Moreover, computational modeling facilitates the exploration of novel MXene-polymer combinations and architectures, guiding experimental efforts toward the synthesis of materials with unprecedented properties or functionalities. By simulating the behavior of nanocomposites at the atomic or molecular level, researchers can uncover underlying mechanisms and design principles that govern their performance, leading to informed decision-making and accelerated materials development cycles (Ohno, Esfarjani, and Kawazoe, 2018).

Overall, future research in the field of MXene-polymer nanocomposites will be characterized by interdisciplinary collaborations, innovative approaches, and transformative applications across various industries. By addressing emerging challenges and exploring new frontiers, researchers aim to unlock the full potential of MXene-based materials for addressing societal needs and advancing technological innovation.

## 7 Conclusion

In conclusion, MXene-polymer nanocomposites hold significant promise in both biomedical fields. These advanced materials offer multifunctional properties, including biocompatibility, tunable surface chemistry, and high adsorption capacity, making them suitable for diverse applications. In biomedicine, MXene-polymer nanocomposites show potential for drug delivery, tissue engineering, biosensors, and medical implants. Ongoing research and development efforts focus on addressing challenges and exploring new frontiers, paving the way for the widespread adoption of MXene-polymer nanocomposites and their transformative impact on healthcare sustainability. In light of the promising potential of MXene-polymer nanocomposites in biomedical applications, it is imperative to emphasize the importance of continued research in this area. By addressing existing challenges, exploring emerging trends, and pushing the boundaries of innovation, researchers can unlock the full capabilities of MXene-polymer nanocomposites. The cost-effectiveness is studied deeply. Therefore, commitment is needed to enhance ongoing collaboration, investment, and exploration in MXene-polymer nanocomposites, as we strive to harness their transformative impact on our society and pave the way for a brighter, more sustainable future.

## Data Availability

The raw data supporting the conclusion of this article will be made available by the authors, without undue reservation.
